# ProstaNet: A Novel Geometric Vector Perceptrons–Graph Neural Network Algorithm for Protein Stability Prediction in Single- and Multiple-Point Mutations with Experimental Validation

**DOI:** 10.34133/research.0674

**Published:** 2025-04-15

**Authors:** Tianjian Liang, Ze-Yu Sun, Rieko Ishima, Xiang-Qun Xie, Ying Xue, Wei Li, Zhiwei Feng

**Affiliations:** ^1^ Department of Pharmaceutical Sciences, Computational Chemical Genomics Screening Center, and Pharmacometrics and System Pharmacology PharmacoAnalytics, School of Pharmacy, National Center of Excellence for Computational Drug Abuse Research, University of Pittsburgh, Pittsburgh, PA 15261, USA.; ^2^ Department of Structural Biology, School of Medicine, University of Pittsburgh, Pittsburgh, PA, USA.; ^3^ Department of Medicine, Center for Antibody Therapeutics, Division of Infectious Diseases, School of Medicine, University of Pittsburgh, Pittsburgh, PA, USA.

## Abstract

Proteins play a critical role in biology and biopharma due to their specificity and minimal side effects. Predicting the effects of mutations on protein stability is vital but experimentally challenging. Deep learning offers an efficient solution to this problem. In the present work, we introduced ProstaNet, a deep learning framework that predicts stability changes resulting from single- and multiple-point mutations using geometric vector perceptrons–graph neural network for 3-dimensional feature processing. For training ProstaNet, we meticulously crafted ProstaDB, a comprehensive and pristine thermodynamics repository, including 3,784 single-point mutations and 1,642 multiple-point mutations. We also created thermodynamic looping for enlarging the limited data size of multiple-point mutation and applied an innovative clustering method to generate a standard testing set of multiple-point mutation. Besides, we identified residue scoring as the most important encoding method in protein properties prediction. With these innovations, ProstaNet accurately predicts thermostability changes for both single-point and multiple-point mutations without showing any bias. ProstaNet achieves an accuracy of 0.75, outperforming existing methods for single-point mutation prediction, including ThermoMPNN (0.63), PoPMuSiC^sym^ (0.66), MUPRO (0.52), and FoldX (0.71). ProstaNet also achieves a 1.3-fold increase in accuracy compared to FoldX for multiple-point mutation predictions. Validated by experiment, 4 out of 5 single-point mutation predictions (80%) and all multiple-point mutation predictions (100%) for HuJ3 mutants were accurate, demonstrating the potential benefits of ProstaNet for protein engineering and drug development.

## Introduction

Proteins play important roles in most biological processes, such as catalyzing biochemical reactions, forming receptors and channels, providing scaffold support, and transporting molecules. Due to proteins’ specific roles in the human body, protein therapeutics are less likely to cause side effects by interfering with normal biological processes [1]. Therefore, protein therapeutics show high potency and can execute more intricate functions to treat complex diseases [2]. Protein thermodynamic stability is one of the important attributes affecting its therapeutic applications and leading to certain aggregation in cellular environment, which causes certain diseases [3]. Meanwhile, protein thermodynamic stability is essential for optimizing the purification, expression, formulation, storage, and structural studies of proteins, which are fundamental aspects in protein therapeutics development [4]. By predicting the effects of mutations on protein stability, a protein with the intended thermodynamic stability can be designed, which is important for biopharmaceutical development. Therefore, accurately predicting how a mutation affects protein stability has become a hotspot in protein engineering.

Experimental methods used to identify mutants that can increase protein stability are often time-consuming, expensive, and labor-intensive. To address this challenge, researchers have leveraged major advancements in computational methods [5–7] to develop tools that efficiently and accurately predict the impact of mutations on protein stability. These tools can identify potential mutants for further experimental testing. These methods are mainly divided into 2 types: physics-based and machine learning-based methods. Physics-based methods rely on sampling the conformation of protein structures and biophysical modeling of amino acid interactions [8–10]. Machine learning-based methods, particularly deep learning-based methods, extract features from protein sequences and structures [11]. Currently, deep learning-based methods are preferred for predicting changes in protein thermodynamic stability upon single-point mutation based on ΔΔ*G* values (change of folding Gibbs free energy) due to their high efficiency and accuracy. For example, ThermoNet [12], a deep 3-dimensional (3D)-convolutional neural network, combines spatially proximate and intricate features with the potential to describe molecular interaction. ThermoMPNN [13] leverages the shared knowledge between sequence recovery and stability optimization tasks by using pretrained PorteinMPNN [14] embeddings as input features for transfer learning. However, there are still some challenges that deep learning-based methods need to overcome to further increase prediction accuracy of protein stability changes upon point mutations. (a) Many thermostability databases collect experimental thermodynamic data that are not comprehensive. The number of experiment-derived thermodynamic data is limited, which affects model performance. The experiment-derived destabilizing mutations account for the majority of these databases. These issues may lead to significant bias during model training and cause them to be prone to overfitting to the training sets [15]. (b) There is only a small amount of software available for predicting protein stability changes of multiple-point mutation. The number of multiple-point mutation data is far less than that of single-point mutation, which hinders the model learning seriously. Also, there is no benchmark testing set for multiple-point mutation data. (c) Since single-point mutation only causes subtle differences in the protein sequence, current deep learning-based methods fall short of representing the changes between a wild-type protein and its mutant counterparts [16]. (d) Recent deep learning methods for learning from protein structures only leverage either geometric or relational information. Geometric information, such as dihedral angles and backbone directions, governs the dynamics and function of the protein. Relational information includes the relationship between protein sequence, residue–residue interactions, and protein properties. Studies show that incorporating both types of information can help methods represent protein 3D structures better [17,18]. (e) The majority of deep learning methods for protein thermostability prediction employ a regression loss function to predict ΔΔ*G* values. While ΔΔ*G* is a continuous value, its direction is crucial for determining the thermostability: a negative ΔΔ*G* indicates a destabilizing mutation, whereas a positive ΔΔ*G* refers to a stabilizing mutation. The regression loss function does not consider the directionality of the value, which does not allow the method to learn meaningful patterns and degrade the method’s performance (see Note S1 for a summary of loss function issues). (f) Feature representation of amino acids is one of the key factors affecting prediction accuracy. However, most deep learning-based methods select features randomly, which may overlook important features or include too many irrelevant ones.

Recently, geometric vector perceptrons–graph neural network (GVP-GNN) [19], has emerged as a deep learning framework that bridges the characteristics of GNNs and convolutional neural networks (CNNs) to learn scalar-valued and vector-valued functions over 3D Euclidean space. The output of GVP-GNN remains unchanged under rotations and reflections in 3D Euclidean space, exhibiting equivariance of invariance. By processing protein graphs containing detailed node and edge features such as backbone directions, dihedral angles, and inter-residue distances, GVP-GNN achieves strong performance on protein properties prediction tasks. Furthermore, by incorporating jumping knowledge to aggregate intermediate representations with the last layer, GVP-GNN is able to highlight subtle changes between wild type and its mutants. Therefore, GVP-GNN has been applied to de novo protein design and protein structural model quality assessment. In both tasks, GVP-GNN exhibited strong performance compared to other deep learning methods. However, GVP-GNN has not yet been applied to protein stability changes prediction. Given its powerful capability, we hypothesize that GVP-GNN will achieve outstanding performance in protein thermodynamic stability prediction.

In this research, we engineered an innovative end-to-end binary classification deep learning model for forecasting changes in protein thermodynamic stability resulting from mutations, employing a specialized type of GNN known as GVP-GNN. GVP-GNN demonstrated superior performance compared to the commonly used GNN variant GCN [20], leading us to build a sophisticated deep learning framework called ProstaNet. ProstaNet ingeniously exploits the strengths of GVP-GNN to assimilate both spatial (geometric) and interactional (relational) characteristics, thereby enhancing prediction precision. To further improve amino acid representation, 7 cutting-edge amino acid encoding methods were applied. We also assessed the contributions of these encoding methods to the thermodynamic stability change prediction. To improve data quality, we constructed ProstaDB, a comprehensive, balanced, and clean thermodynamics database that composes both single-point and multiple-point mutation for training ProstaNet. We used the single-point mutation to pretrain and multiple-point mutation to fine-tune the ProstaNet model. To augment the multiple-point mutation data, we introduced a data augmentation technique called thermodynamic looping (TL). We also applied an advanced clustering method to make the distribution of data similar between training and testing sets. Our results show that with advanced architecture and a comprehensive database, ProstaNet outperformed commonly used methods in predicting protein thermodynamics stability changes especially upon multiple-point mutation. Experimental results confirmed that 4 out of 5 single-point mutation predictions and all 4 multiple-point mutation predictions for HuJ3 mutants were accurate. These results underscore that ProstaNet is reliable in protein thermostability prediction.

## Results

### An overview of ProstaNet

**Fig. 1.**
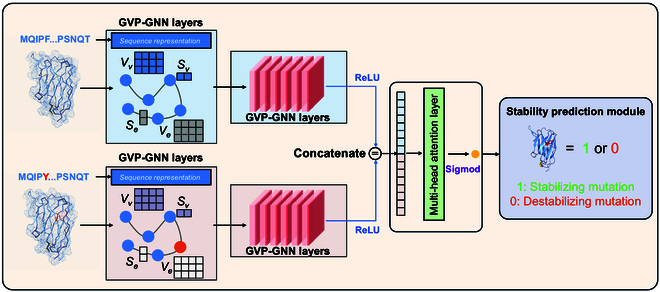
The architecture of ProstaNet. The 3D structures of wild-type and mutant proteins are converted into structure graphs. Their sequences are embedded by advanced amino acid encoding methods and severed as scalar vector of the nodes. Structure graphs are passed to GVP-GNN layers. The feature vectors of wild-type and mutant proteins are concatenated. Then, pass to the multi-head attention layer and generate the “0” or “1” as the final output.

ProstaNet leverages the novel deep learning framework GVP-GNN to learn scalar and vector features over 3D Euclidean space. This method requires a 3D structure of either wild-type or mutant protein. ProstaNet generates a 3D structure for the mutant protein from the wild-type structure using Rosetta Fast Relax. The 3D structures of the wild-type and mutant proteins are converted into 2 structural graphs. The amino acid encoding methods process the protein sequences to represent the features of the amino acids, which are concatenated into the node scalar features ($S_{\nu }$) within the graphs. Amino acids orientation is used as the node vector features ($V_{\nu }$). The distance between 2 atoms is used as the edge scalar feature ($S_{e}$) and the orientation of the link between 2 atoms is used as the edge vector feature ($V_{e}$). GVP-GNN layers extract the feature vectors from these 2 graphs. These vectors are concatenated and passed through multi-head attention layers to capture the difference between wild-type and mutant vectors. ProstaNet performs binary classification based on the range of ΔΔ*G* to determine whether a mutation is stabilizing or destabilizing. An output “1” indicates a stabilizing mutation and “0” indicates a destabilizing mutation (Fig. 1).

### ProstaDB: A robust protein thermostability database

**Fig. 2.**
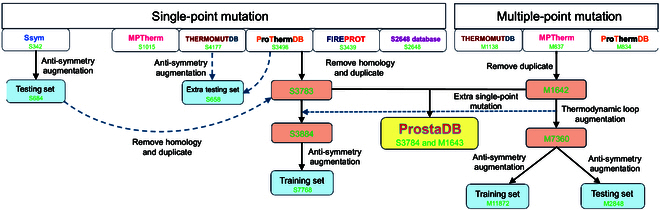
Pipeline of generating ProstaDB, training, and testing sets. The data in the S3783 dataset that are homologous with the data in the testing set are removed. PSI-BLAST is used to identify the homology proteins. The data in the extra testing set are collected from ThermoMutDB and ProThermDB. The single-point mutations derived from TL augmentation are added to the single-point mutation training set. “S” in “S3784” means single-point mutation data, and “3784” means the number of the unique data. “M” in “M1643” means multiple-point mutation data.

The quantity of training data is a crucial factor influencing the learning capability of a deep learning method. A large amount of training data enables the model to better identify data patterns and make more accurate predictions. However, none of the commonly used protein thermodynamic databases provide comprehensive data. We created a comprehensive thermodynamic database called ProstaDB that contains 3,784 single-point and 1,643 multiple-point mutation data (Fig. 2 and Note S4).

To solve the problem of limitation of multiple-point mutation data (see Note S3 for issues of current data augmentation methods), we introduced a data augmentation method called TL to augment the M1642 data, which was based on energy conservation (Fig. S2 and Note S5). If both variant A and variant B share the same wild type, experimental data indicate that variant A has a higher energy state, while variant B has a lower energy state compared to the wild type. To ensure energy conservation in the cycle involving the wild type, variant A, and variant B, the ΔΔ*G* value for the mutation from variant A to variant B should be less than 0 kcal/mol. With TL, the multiple-point mutation data augment from 1,642 (M1642) to 7,360 (M7360). TL generated 101 single-point mutation data, which was added to the S3783 dataset and formed the S3884 dataset. The S^sym^ dataset [21] was applied as the testing set, as it is the benchmark for the protein stability changes upon single-point mutation. Currently, multiple-point mutation does not have a benchmark testing set. Thus, we generated the testing set by splitting the data from the M7360 dataset. The combination of mutation types affects the generalization of models in predicting multiple-point mutation. We introduced a clustering method to cluster the different combinations of mutation types into several groups. Then, let the distribution of different groups be similar between the training and testing sets (Fig. S3). The M7360 database was split into training and testing sets with a ratio of 8:2.

To further enlarge all the datasets and balance stabilizing and destabilizing data within the dataset, we implemented anti-symmetry-based data augmentation (thermodynamic reversibility [TR]). If protein Y is generated by mutating amino acid X to Y, it is called a direct mutation. Conversely, if protein X is generated by mutating amino acid Y to X, it is called a reverse mutation. The ΔΔ*G* value for the mutation from protein X to Y is the inverse of that from protein Y to X. TR produced a total of 7,768 data points (S7768) as training and validation sets for single-point mutation. The S^sym^ dataset (684 data points) is the testing set and 658 data points (S658) are the extra testing set for single-point mutation. There are 11,872 (M11872) data points as the training set and 2,848 (M2848) data points as the testing set for the multiple-point mutation.

### Impact of training strategies on ProstaNet performance

**Fig. 3.**
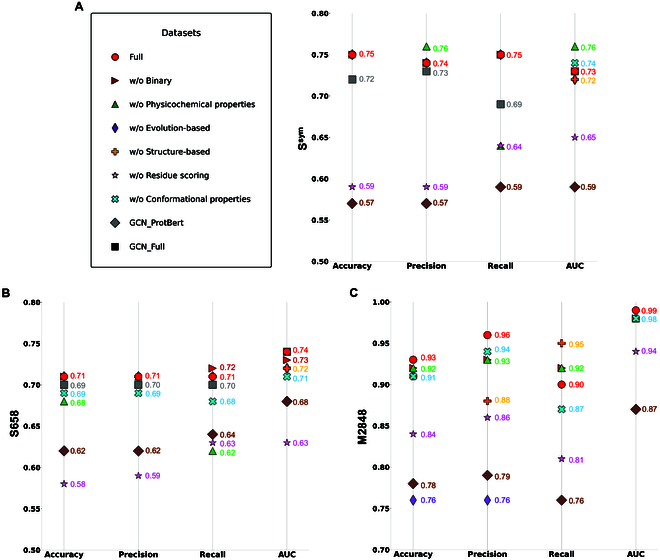
Results of ProstaNet with different encoding methods and GCN model in protein thermostability prediction upon mutations. (A to C) Performance of ProstaNet with different combinations of amino acid encoding methods and GCN models in predicting S^sym^ (A), S658 (B), and M2848 (C) testing sets. The performance of the “GCN_Full” model in predicting M2848 is as follows: accuracy, 0.59; precision, 0.58; recall, 0.67; and AUC, 0.63. Since all the values for “GCN_Full” are below 0.65, we do not include its results in (C) to improve clarity.

We employed 6 novel categories of amino acid encoding methods—binary, physicochemical properties, evolution-based, structure-based, residue scoring, and protein conformational properties—to represent the amino acids in the protein graph. Six amino acid encoding methods concatenate to represent amino acids in the protein graph. Since GCN is one of the representatives in GNN, we built a GCN-based model (Fig. S4 and Note S2) and compared it with ProstaNet. We called models using S7768 to pretrain and M11872 to fine-tune ProstaNet and GCN-based as ProstaNet_Full and GCN_Full, respectively. Regarding the results, ProstaNet shows better performance than the GCN-based model in predicting protein thermostability changes, especially for multiple-point mutation (Fig. 3A to C). This may be because protein multiple-point mutation is more complex than single-point mutation, which involves more geometric and relational changes between amino acids. Because GVP-GNN can utilize both geometric and relational features, it captures more pattern from the data and handles complicated tasks.

**Fig. 4.**
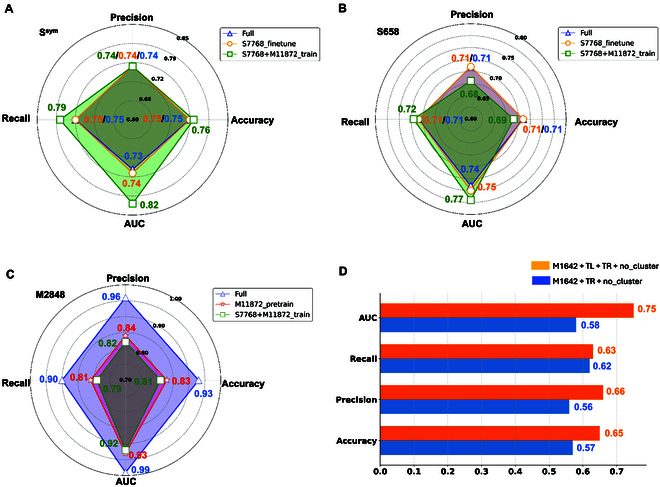
Results of different models in protein thermostability prediction upon mutations. (A to C) Performance of training with different orders of dataset, combined training with single-point and multiple-point mutations data, and training with or without TL augmentation datasets on S^sym^ (A), S658 (B), and M2848 (C) dataset. (D) Performance of fine-tuning with or without TL augmentation data on M2848 dataset. Because ProtBert encodes amino acids into high-dimensional space features, the features are not explicitly predefined. Therefore, ProtBert was not a suitable encoding method for ProstaNet, and we only applied ProtBert to extract features for the GCN-based method.

To study the impact of training strategies on ProstaNet performance, we compared training using different orders of single-point and multiple-point mutations data, with and without TL augmentation, and with and without clustering methods to generate the testing set. The performance was then evaluated on the testing sets. Figure 4A to C shows that fine-tuning the model with multiple-point mutation data achieves higher accuracy in predicting thermostability changes than pretraining the same data. This is due to the multiple-point mutation being formed by several single-point mutations; hence, the relationship between protein structure and thermostability learned from single-point mutation data enables the model to better recognize patterns in multiple-point mutation data, improving its performance. The model fine-tuned by S7768 shows similar results with using S7768 to pretrain the model, which indicates that the patterns of multiple-point mutation data learned by the model did not assist it to learn the patterns of single-point mutation. Figure 4A and B shows that the combined S7768 and M11872 datasets to train ProstaNet have a slightly higher accuracy in predicting the S^sym^ dataset compared with ProstaNet_Full. However, the accuracy of the S7768 + M11872 model in predicting the S658 testing set is lower than ProstaNet_Full in predicting the S658 testing set, indicating that combining the 2 training sets to train ProstaNet has reduced the model’s generalization ability. The S7768 + M11872 model performs worse in predicting multiple-point mutations compared to ProstaNet_Full (Fig. 4C). Consequently, using single-point mutation to pretrain model and then using multiple-point mutation to fine-tune is a good training strategy for ProstaNet.

Figure 4D indicates that the model trained on the dataset with TL augmentation (M1642 + TL + TR + no_cluster) has a higher accuracy than the model trained on the dataset without TL augmentation (M1642 + TR + no_cluster). TL augmentation enlarged the data size, leading the model to better recognize the patterns of the data, and improved the generalization of the model, which enhanced the performance of the model in predicting thermostability changes upon multiple-point mutation. Compared with ProstaNet_Full, M1642 + TL + TR + no_cluster showed lower accuracy. Figure S5A (second and third rows) shows that without the clustering method, the mutation types of training and testing sets are markedly different compared to when the clustering method is applied. The testing set that was not divided by the clustering method had several mutation types that were not present in its training set. Some of these mutation types also had high occurrence frequency in the testing set, which leads the model to not learn the pattern of these mutation types and lower its generalization (Fig. S5B). These results also indicate that the mutation-type combination distribution between training and testing sets has a huge impact on protein thermostability prediction; it is important to have training and testing sets that have a similar distribution of mutation-type combinations. In general, the TL augmentation technique and the novel clustering method can increase the quality of data and improve the performance of ProstaNet.

### Contribution of amino acid encoding methods

The representation of amino acids is one of the fundamental factors affecting the efficacy of a deep learning method. In this study, we studied the effect of these amino acid encoding methods on protein thermostability prediction. We also tested the novel protein language models, ProtBert. To evaluate the importance of each feature, an ablation study was conducted. According to Fig. 3A and B and Tables S5 and S6, without residue scoring encoding, the model has drastically impaired performance in predicting both S^sym^ and S658 datasets. The model without one-hot, evolution-based, physicochemical, structure-based, and protein conformational features still achieves similar and strong performance on S^sym^ and S658 datasets, suggesting that these features do not substantially affect the quality of the learned representation. Combining the results of S^sym^ and S658 datasets, all encoding combined and without residue scoring methods have a higher accuracy in predicting both datasets. In predicting the M2848 dataset, all encoding combined method has the highest accuracy among the others (Fig. 3C and Tables S7 and S8). The without evolution-based encoding method shows notably weak performance, which illustrates that evolution-based encoding has a critical effect in representing amino acid features for predicting thermostability changes upon multiple-point mutation. The without residue scoring encoding method still shows lower accuracy compared to other methods except the without evolution-based encoding method. Overall results demonstrate that the residue scoring encoding method is one of the key amino acid representation methods. Besides, all these 6 encoding methods combined is more effective to represent amino acids in predicting protein thermostability changes on mutations.

As shown in Fig. 3A to C, the accuracy for the GCN_ProtBert method is lower than that for GCN_Full in predicting single-point mutation. However, the accuracy for GCN_ProtBert is higher than that for GCN_Full in predicting multiple-point mutation. The result indicates that GCN_ProtBert causes overfitting. This overfitting is likely due to insufficient single-point mutation training data to adequately learn the high-dimensional features encoded by ProtBert. When the data size is large in the multiple-point mutation dataset, the GCN_ProtBert has better performance. In this case, it is better to apply explicitly predefined low-dimensional amino acid encoding methods instead of high-dimensional ones when data size is small.

### Comparison with state-of-the-art methods

**Fig. 5.**
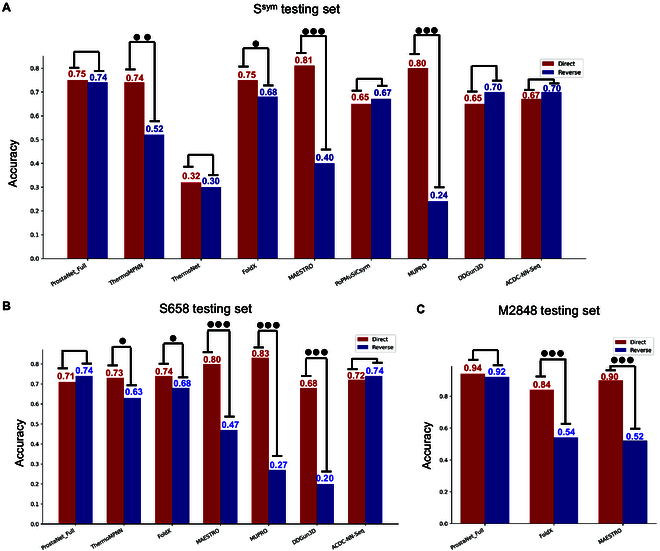
The accuracy of ProstaNet and different protein thermostability predictors across several testing sets. The direct and reverse accuracy of ProstaNet and different protein thermostability predictors. (A) The accuracy upon the S^sym^ testing set. (B) The accuracy upon the S658 testing set. (C) The accuracy upon the M2848 testing set. We calculated the difference between direct and reverse accuracy by subtracting reverse accuracy by direct accuracy. “●” indicates 0.05 < difference ≤ 0.1. “●●” indicates 0.1 < difference ≤ 0.2. “●●●” indicates difference > 0.2.

ProstaNet was compared to state-of-the-art protein thermostability change predictors upon single-point and multiple-point mutations. A summary of these predictors is provided in Table S1. To evaluate the performance of each method with respect to the protein thermostability changes from direct and reverse mutations, the direct and reverse mutations in S^sym^, S658, and M2848 were separated into 2 distinct testing sets. The prediction accuracy on these sets was used to evaluate model performance. High and comparable prediction accuracy for both direct and reverse mutations indicates that a model is an unbiased predictor with a powerful performance. Figure 5 demonstrates that ProstaNet_Full achieves high prediction accuracy for both direct and reverse mutations, suggesting that it does not exhibit bias toward either mutation type.

ProstaNet_Full demonstrates a strong performance compared to state-of-the-art methods, particularly achieving the highest prediction accuracy for reverse mutations. MUPRO [22], MAESTRPO [23], and ThermoMPNN [13] are not-antisymmetric predictors. In protein thermostability changes upon single-point mutation prediction, although MUPRO and MAESTRO surpass ProstaNet_Full in predicting direct mutations, they exhibited a significant bias toward destabilizing mutations. Due to training on the Megascale dataset, ThermoMPNN showed less bias compared to MUPRO and MAESTRO. On the other hand, the antisymmetric predictors ThermoNet, FoldX [24], PoPMuSiC^sym^, and ACDC-NN-Seq [25] do not show strong bias in predicting both S^sym^ and S658 testing sets. DDGun3D [26] does not show significant bias in predicting S^sym^ but does for the S658 testing set. Among these, FoldX outperforms the other antisymmetric predictors and shows little higher accuracy than ProstaNet_Full in direct mutation prediction of single-point mutation, while ThermoNet showed comparatively poorer performance for both direct and reverse mutations in the S^sym^ testing set (Fig. 5A and B). ProstaNet also demonstrates outstanding performance in protein thermostability change upon multiple-point mutation (Table S4). FoldX and MAESTRO exhibit obvious bias in predicting multiple-point mutation. Combining the results from all testing sets. ProstaNet_Full demonstrates strong performance compared to state-of-the-art methods, especially in predicting protein thermostability change upon multiple-point mutation (Fig. 5C).

### Experimental validation on HuJ3

**Fig. 6.**
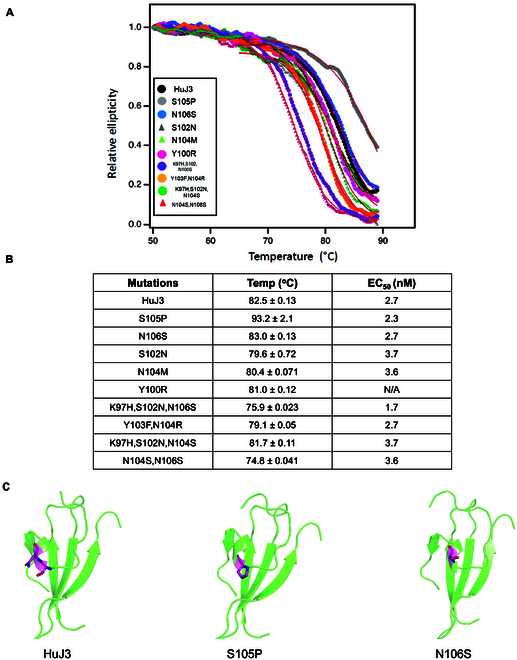
CD spectroscopy and ELISA results. (A) Thermal denaturation curve of mutants that we predicted. (B) The melting temperature and EC_50_ of mutants we predicted. (C) 3D structures of HuJ3 wild type, S105P mutant, and N106S mutant.

To further validate the single-point mutation trained ProstaNet model accuracy, we used it to predict the stability changes of humanized J3. Humanized J3 is a nanobody that binds with gp120 against HIV-1 and showed profound neutralization ability, leveraging the advantages of nanobody [27,28]. Our laboratory previously identified 5 important single-point mutations on HuJ3. We applied the pretrained model to predict the stability changes of these mutations. Since HuJ3 does not have a crystal structure, AlphaFold2 was utilized to obtain the HuJ3 3D model by its sequence. Rosetta Fast Relax was used to relax wild-type HuJ3 and generated 5 variants. The 3D structures were converted into protein graphs and input to the pretrained model. Our model predicted that three of the mutations (S105P, N106S, and N104M) can increase HuJ3 thermostability. Circular dichroism (CD) spectroscopy results (Fig. 6A) showed that mutation S105P increases HuJ3 melting temperature, *T*_m_, N106S has the same *T*_m_ as (or slightly higher *T*_m_ than) HuJ3, and the other 3 mutations have lower HuJ3 thermostability, which means that 4 of the 5 predictions match the experimental data. Among the 2 potential mutations, S105P can considerably increase HuJ3 stability. Based on the structure analysis, S105 is located in a loop of HuJ3; proline often reduces flexibility in the loop and decreases entropy in the unfolded state. Hence, S105P mutation makes the protein less dynamic and more resistant to denaturation at high temperatures. N106 is also located in a loop of HuJ3; asparagine has a longer side chain than serine. The mutation from N to S could reduce local conformational entropy in the unfolder state and relieve steric clashes to make local geometry more stable (Fig. 6C).

Multiple-point mutation fine-tuned models were also applied to predict protein stability changes of HuJ3 on multiple-point mutation. The 4 potential multiple-point mutations identified by our laboratory were used to validate the model. The fine-tuned model predicted that all 4 mutations could not increase the stability of HuJ3. These predictions were also proved by the CD spectroscopy results; all mutants have a lower *T*_m_ than HuJ3, which means that all predictions match the experimental data. All these results showed that both single-point and multiple-point mutation models could predict stability changes of proteins during mutations accurately. Meanwhile, we compared the thermostability of these mutations with their binding affinity with gp120, the results of which were identical to those from previous experiments performed by our laboratory (Fig. 6B). We found that there is no direct correlation between protein thermostability and its binding affinity with the target. For Y100R, it cannot reach the maximum effect for inducing biological effect with gp120; thus, it does not have half maximal effective concentration (EC_50_) value. Combined with the thermostability result, we can exclude the deduction of thermostability to be the reason why Y100R cannot test the EC_50_ value.

## Discussion

In this study, we applied the novel neural network GVP-GNN to build an advanced binary classification deep learning method called ProstaNet to predict protein thermostability changes upon single-point and multiple-point mutations. Leveraging the ability of GVP-GNN to handle scalar and vector features in protein graphs allows ProstaNet to learn from geometric information at nodes and edges without reducing such information to scalars. This enables ProstaNet to capture more informative patterns of protein properties from protein structures. We also constructed ProstaDB, a comprehensive, balanced, and clean thermodynamics database that composes both single-point and multiple-point mutations. We applied a novel data augmentation method to enlarge multiple-point mutation data size and utilized a clustering method to generate an effective multiple-point mutation testing set. Additionally, we applied several advanced amino acid encoding methods to extract amino acid features and identified the importance of each feature in amino acid representation. Our results indicated that the residue scoring encoding method substantially enhanced feature representation, suggesting that it may be used as the key feature to predict protein properties. Besides, the position specific scoring matrix (PSSM) encoding method could enhance feature representation in multiple-point mutation data, indicating that it may play a key role in predicting protein properties when evolving multiple-point mutation. With its powerful architecture and advanced encoding methods, ProstaNet can extract more informative patterns from protein graphs. The results prove that with a balanced, larger, and non-redundant dataset, ProstaNet shows strong performance and reduced bias compared with other predictors. ProstaNet demonstrates its potential for accurately predicting protein thermostability changes in both single-point and multiple-point mutations.

Despite ProstaNet achieving high accuracy in predicting protein stability changes, it has certain limitations. First, the limited training data hinders ProstaNet from fully learning the patterns from protein structures and restricts its performance. The single-point mutation data currently do not have a reliable data augmentation method. Besides, the ΔΔ*G* values of mutations in current thermodynamic databases are not tested under consistent conditions (pH and temperature), which may introduce noise to the model. Secondly, the predictions of ProstaNet cannot fully match the experimental validation; this is because ProstaNet is trained on protein crystal structures; it tends to achieve high accuracy specifically for these structures. The proteins that ProstaNet used to predict did not have crystal structures and their 3D structures were obtained from AlphaFold2. Although AlphaFold2 predicts proteins 3D structures accurately, the geometric and relational information of amino acids on predicted proteins still have some differences from the native one. ProstaNet learns the pattern from amino acids’ geometric and relational information; the difference from predicted and crystal structures will lower the accuracy of ProstaNet. In most cases, crystal structures are not available, which limits the application of our method. Thirdly, because ProstaNet identifies the patterns based on structural information in protein graphs, the quality of the mutant 3D structures strongly affects its performance. Currently, we apply Rosetta Fast relax to generate 3D structures of mutant proteins. However, Rosetta Fast relax only searches the local conformational space around the starting structure and does not do extensive refinement. In reality, certain mutations can induce considerable structural changes that might exceed the capabilities of Rosetta’s Fast Relax module for accurate simulation. Consequently, the modeled structures of these mutated proteins may not reliably mirror their natural conformations. This limitation hinders the method’s ability to extract genuine patterns from the mutant structures accurately.

Currently, much of the existing research in protein stability prediction focuses on the regression task of predicting ΔΔ*G* values. They apply mean square error as the loss function. However, the mean square error only measures the magnitude of errors between the predicted and actual ΔΔ*G* values and omits the sign (positive and negative) of the error. Due to lack of training data, using the regression task will increase the difficulty in predicting protein stability changes accurately. For instance, ThermoNet has relatively high Pearson correlation coefficients (*R*) for ΔΔ*G* prediction on S^sym^ (0.58 and 0.59 for direct and reverse mutations, respectively) but demonstrates lower classification accuracy. Conversely, MAESTRO exhibits lower *R* values (0.52 and 0.32 for direct and reverse mutations, respectively) for testing on S^sym^ but achieves higher classification accuracy. This means that optimizing for ΔΔ*G* prediction does not necessarily translate to improved stability classification performance. Because of the limited availability of high-quality training data, ProstaNet prioritizes improving classification accuracy to ensure reliable identification of stabilizing mutations. However, ΔΔ*G* prediction is still important in the broader field of protein stability optimization. As part of future work, we plan to refine ProstaNet to integrate ΔΔ*G* prediction while maintaining its classification performance.

In the future, we will also create a new database that includes mutation data for both crystal and modeled structures. Meanwhile, we will classify the mutation data based on its testing environment to let the users select the data based on their needs. This will greatly increase the number of mutation data. Besides, it will allow our method to learn from both crystal and modeled structures to broaden the scope of the method’s application. To increase the quality of mutant structures, we will apply more powerful modeling techniques, for example, AlphaFold2, to generate 3D structures of mutant proteins.

## Methods

### Dataset description

#### ProstaDB

We combined all protein thermodynamics single-point mutation data from the ProThermDB [29], S2648 database [30], ThermoMutDB [31], MPTherm [32], and FireProtDB [33] databases, totaling 14,777 data points (S14777). Multiple-point mutation data were collected from ThermoMutDB, MPTherm, and ProThermDB, totaling 2,609 data points (M2609). All these databases were measured experimentally and expressed quantitatively as ΔΔ*G*, Δ*T*_m_, or both values. First, we removed duplicate data from each database. Since most protein stability changes in ProThermDB and MPTherm are expressed quantitatively as Δ*T*_m_, with some as ΔΔ*G*, we selected data containing either ΔΔ*G* or Δ*T*_m_ values from these databases. For all other databases, where stability changes are expressed quantitatively as ΔΔ*G*, only data with ΔΔ*G* values were selected. Proteins with high sequence similarity are referred to as homologous data. Based on the previous research [34], different single-point mutation in homologous proteins is not necessarily independent. Homologous proteins present in both the training and testing sets can lead to data leakage, which negatively impacts the generalization of the method. The multiple-point mutation is the combination of different mutation types. Because the extreme lack of data and the combinations of mutation types have more impact on the stability changes of multiple-point mutation, we only remove the duplicate data within the M2609 database. To eliminate data leakage, the duplicated and homologous data within S14777 and M2609 were removed. Also, the data in S14777 that are homologous with data in S^sym^ were also removed. Following the works of Jing et al. [35], the blastp program was used to identify and exclude homology data (BLAST e-value < 0.001). Our thorough collection and cleaning data of the S14777 and M2609 datasets left 3,783 unique data for single-point mutation and 1,642 unique data for multiple-point mutation. These 2 datasets were combined to form the comprehensive database called ProstaDB (Table S2).

#### Training and testing datasets for ProstaNet

M1642 used the TL method to augment the data to 7,360 unique data points. The augmentation method generated 101 single-point mutation data points. These data points were combined with the S3783 dataset forming the S3884 dataset for training. The gold standard testing set S^sym^ with 342 data points was used as one of the testing sets. The single-point mutation data from ThermoMutDB and ProThermDB whose Protein Data Bank (PDB) number was presented in S^sym^ but the mutation did not show in S^sym^ were collected as the extra testing set with 329 unique data points. The TR was applied to swap the wild type and mutant to augment the single-point mutation data, resulting in 7,768 unique single-point mutation data points for pretraining the ProstaNet and 1,342 data points for testing pretrained models. Since there is no gold standard testing set for multiple-point mutation, the M7360 dataset was split into an 8:2 ratio for training and testing. The TR applied to augment the data resulted in 11,872 multiple-point mutation data points for fine-tuning the pretrained ProstaNet. There were 2,848 multiple-point mutation data points for testing fine-tuned ProstaNet. For all, the training data were split into 80% for training and 20% for validation.

#### Modeling mutant structures

For each mutation in ProstaDB and S^sym^, the protein has a crystal structure designated as protein X and the other as protein Y. The 3D structure of protein X was collected from the PDB and relaxed by the Rosetta Fast Relax using Rosetta all-atom energy function ref2015. Based on the input structure, the backbone heavy atoms were added coordinate constraints to prevent large-scale conformational shift from the starting structure. To generate the 3D structure of protein Y, the same Rosetta Fast Relax protocol was also employed to mutate protein X to its corresponding protein Y. The Rosetta resfile was applied to specify mutation X to Y.

#### Novel clustering method and generation of the multiple-point mutation testing set

Regarding our novel clustering method, the mutation-type combination of each mutation data was separated into single mutation types. We refer to the original (or parent) amino acids as “Mutation from” amino acids, and the amino acids they mutate into as “Mutation to” amino acids. We have 298 mutation types in total. One-hot encoding was utilized to encode each mutation data. Since the relationship between different combinations of mutation types is nonlinear, t-distributed stochastic neighbor embedding (t-SNE), a nonlinear technique that focuses on preserving local pairwise similarities, is more suitable for our task. Therefore, we used t-SNE to perform dimension reduction. Because we did not know the number of clusters needed and the cluster shapes were arbitrary, we used DBSCAN, which is capable of identifying clusters of arbitrary shapes and varying sizes. Therefore, we used DBSCAN to cluster different combinations of mutation types in M7360 into 16 groups. Then, we calculated the ratio of data in different groups. The number of data selected from each group followed their data ratio and the wild-type proteins were chosen randomly (Table S3). To ensure that the training and testing sets do not contain wild-type proteins with the same sequence, all mutation data for the selected wild-type proteins will be included in the testing set. The data size in group 9 was small, and we can only select the wild-type proteins with the smallest data size, but the number of the data was still larger than the data we should have taken from group 9. Since the mutation data in groups 11 and 12 belonged to a few wild-type proteins, if we included one of the wild-type proteins, the number of data we took would be far larger than the number we should take. Thus, these 2 groups could not collect enough data into the testing set.

#### Training models

We chose the specific encoding methods from each category of amino acid encoding methods based on the results from Jing et al. [35] to represent amino acids in the protein graph. We used one-hot encoding for binary features, Atchley factor encoding for physicochemical properties features, PSSM encoding for evolution-based features, Micheletti potential encoding for structure-based features, 20 metrics from the Rosetta scoring function for residue scoring features, and dihedral angle for protein conformational properties features. During training, we used binary cross-entropy as the loss function and 5-fold cross-validation to validate models. The S7768 dataset was used to pretrain ProstaNet, with 3 GVP-GNN layers, 2 GVPs used in message function, 4 GVPs used in feedforward function, hidden layers with dimension 182, batch size 64, dropout 0.6, learning rate 0.0001, and Adam optimizer with weight decay 0.001. To fine-tune a single-point mutation trained model on M11872, we tuned the parameters of GVP-GNN layers, with learning rate 0.001, weight decay 1 × 10^−6^, and dropout 0.4. All the models were trained on a single l40s GVP with 16 dedicated CPUs.

#### Variants of ProstaNet

We have generated different versions of the ProstaNet model in identifying the impact of training strategies on ProstaNet. To verify the function of TL augmentation, the S7768 dataset was used to pretrain ProstaNet. The pretrained model was then fine-tuned by different multiple-point mutation data. To generate the fine-tuned data of M1642 + TR + no_cluster, we split the M1642 dataset into 80% for training and 20% for testing. We randomly selected 328 data points from the M1642 dataset as the testing set and the remaining data as the training set. TR augmentation enlarged both training and testing tests, then used to fine-tune the pretrained model. To obtain fine-tuned data of M1642 + TL + TR + no_cluster, we split the TL augmented M7360 dataset into training and testing sets using the ratio 8:2. We randomly selected 1,484 data points from the M7360 dataset and the remainder as the training set. After TR augmentation, the training set was used to fine-tune the pretrained model. As for testing the impact of using M11872 to pretrain and S7768 to fine-tune ProstaNet, we applied the M11872 dataset to pretrain ProstaNet then fine-tune it by S7768. For testing the S7768 + M11872 directly trained model, we combined S7768 and M11872 datasets to train ProstaNet.

#### Comparison with state-of-the-art methods

We evaluated ProstaNet with state-of-the-art methods and compared the value of prediction accuracy of direct mutations, reverse mutations, and average accuracy. All testing sets were split into direct mutation and reverse mutation testing sets. The average accuracy was the average of the accuracy of direct and reverse mutations. Because the code provided by ThermoNet on GitHub could not run and PoPMuSiC^sym^ only has a web server, the S^sym^ results of ThermoNet and PoPMuSiC^sym^ came from a paper [36], and they could not be tested on the S658 testing set. The DynaMut2 [37] could be used to predict multiple-point mutation. However, it only has web servers and application programming interface (API) did not work. Thus, we did not test the performance on the M2848 testing set. The results of other predictors were generated by running the programs using the code provided on the papers.

#### Protein thermostability testing

To generate the mutated variants, the protein sequences were reverse-translated into corresponding DNA sequences and synthesized by integrated DNA technologies (IDT). These antibody genes were cloned into the pcomb3X plasmid and transformed into competent top 10 *Escherichia coli* cells. The transformants were spread onto 2YT agar plates containing ampicillin and incubated overnight at 37 °C. Individual colonies were selected and cultured, and the plasmids were extracted and sequenced using Sanger sequencing (Genewiz). Verified plasmids were then re-transformed into competent top 10 *E. coli* for expression. Cultures were grown at 37 °C with shaking for 8 h, followed by protein induction with 1 mM isopropyl-β-D-thiogalactopyranoside at 30 °C for 16 h. The proteins were purified using Ni-NTA Agarose columns (QIAGEN), and their purity was assessed via sodium dodecyl–polyacrylamide gel electrophoresis analysis. CD experiments were performed using the proteins at 7 μM in a 20 mM Tris buffer, pH 7.4. Protein thermal stability was assessed by measuring ellipticity change at 226 nm at a heating rate 0.2 °C/min, using a JASCO J810 spectrometer. For each protein, thermal melting temperature, *T*_m_, was determined by fitting the temperature-dependent data using Igor Pro software (WaveMetrics, Portland, OR).

## Data Availability

The source code of all 2 in-house software is freely available on GitHub under Apache-2.0 license. The code and raw data can be downloaded on GitHub (https://github.com/NikoBelice/ProstaNet). All the PDB that was used in the paper is available in Zenodo (https://doi.org/10.5281/zenodo.14658434).

## Supplementary Materials

Supplementary 1 Notes S1 to S9 Figs. S1 to S5 Tables S1 to S8
