# Supplementary material for: ProstaNet: A Novel Geometric Vector Perceptrons–Graph Neural Network Algorithm for Protein Stability Prediction in Single- and Multiple-Point Mutations with Experimental Validation
Source: Research (Wash D C). 2025 Apr 15;8:0674. doi: 10.34133/research.0674 (PMC11997553; doi:10.34133/research.0674)
Supplement: Supplementary 1 — Notes S1 to S9 Figs. S1 to S5 Tables S1 to S8 [file research.0674.f1.zip › FigureS5.pdf]

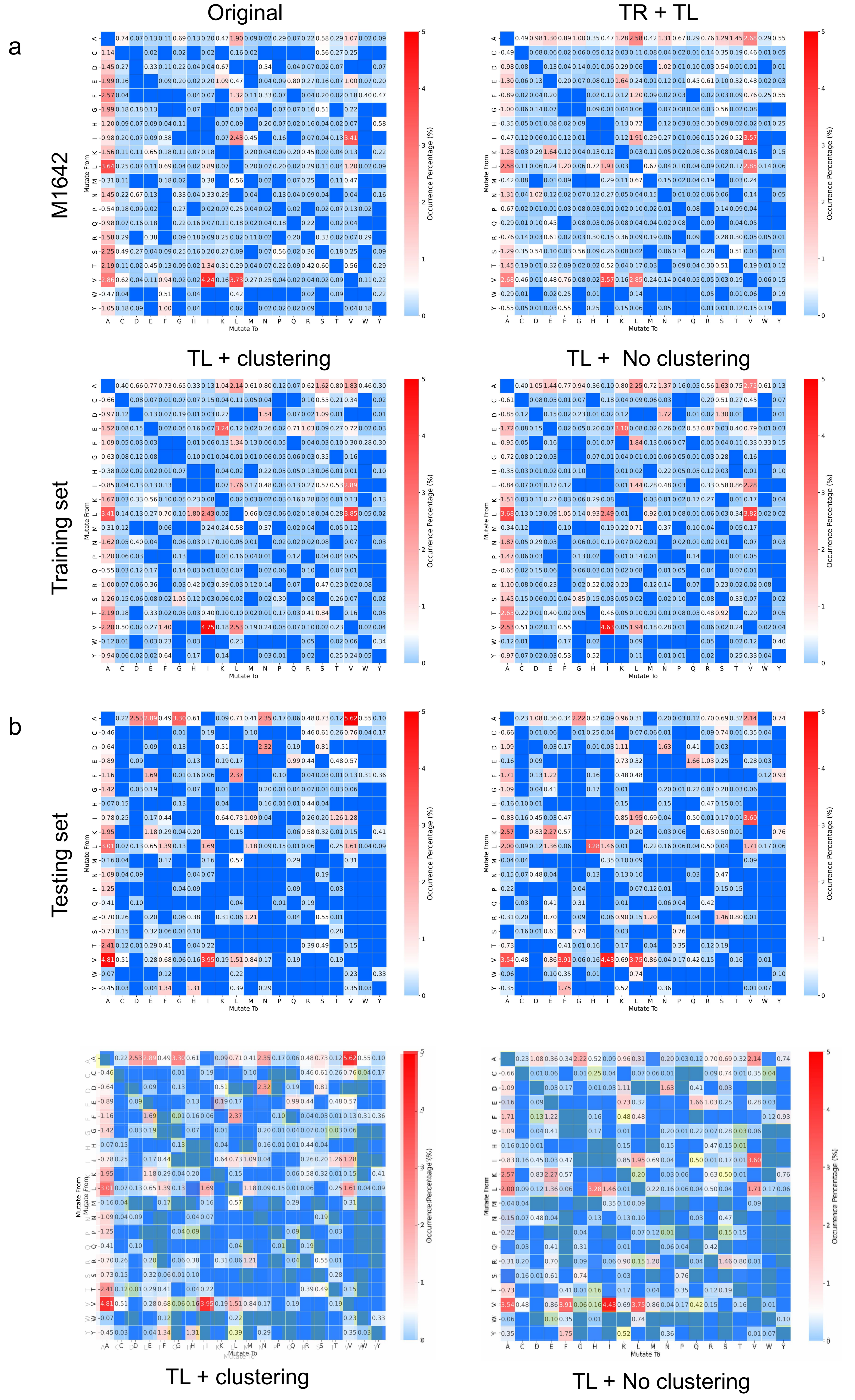
a
Original
TR + TL
M1642
TL + clustering
TL + No clustering
Training set
b
Testing set
TL + clustering
TL + No clustering
Mutate From
Mutate To
Occurrence Percentage (%)
