# Supplementary material for: ProstaNet: A Novel Geometric Vector Perceptrons–Graph Neural Network Algorithm for Protein Stability Prediction in Single- and Multiple-Point Mutations with Experimental Validation
Source: Research (Wash D C). 2025 Apr 15;8:0674. doi: 10.34133/research.0674 (PMC11997553; doi:10.34133/research.0674)
Supplement: Supplementary 1 — Notes S1 to S9 Figs. S1 to S5 Tables S1 to S8 [file research.0674.f1.zip › ProstaNet_sp_research.docx]

**Supplemental Materials**

**ProstaNet: A Novel GVP-GNN Algorithm for Protein Stability Prediction in Single and Multiple Point Mutations with Experimental Validation**

**Tianjian Liang^1†^, Ze-Yu Sun^1†^, Rieko Ishima^2^, Xiang-Qun Xie^1*^, Ying Xue^1*^, Wei Li^3*^, Zhiwei Feng^1*^**

^1^Department of Pharmaceutical Sciences, Computational Chemical Genomics Screening Center, and Pharmacometrics & System Pharmacology PharmacoAnalytics, School of Pharmacy; National Center of Excellence for Computational Drug Abuse Research, University of Pittsburgh, Pittsburgh, Pennsylvania 15261, United States

^2^Department of Structural Biology, School of Medicine, University of Pittsburgh, Pittsburgh, Pennsylvania, United States

^3^Department of Medicine, Center for Antibody Therapeutics, Division of Infectious Diseases, School of Medicine, University of Pittsburgh, Pittsburgh, Pennsylvania, United States

*Address correspondence to: [Sean.Xie@pitt.edu](mailto:Sean.Xie@pitt.edu) (X.X.); [yix49@pitt.edu](mailto:yix49@pitt.edu) (Y.X.); [liwei171@pitt.edu](mailto:liwei171@pitt.edu) (W.L.); [zhf11@pitt.edu](mailto:zhf11@pitt.edu) (Z.F.)

^†^The authors wish to be known that, in their opinion, the first two authors should be regarded as joint first authors.

**Supplementary Notes**

**1. Task and metrics selection**

Many widely used protein thermostability change predictors take protein stability change upon mutations as the regression task. They apply mean square error as the loss function and predict the values of ΔΔG of mutations. The direction (positive and negative) of ΔΔG will determine if the mutation is a stabilizing mutation or a destabilizing mutation. However, the mean square error only measures the magnitude of errors by squaring the difference between the predicted and actual ΔΔG values, which remove the sign (positive and negative) of the error. Due to lack of training data, using the regression task will increase the difficulty in predicting protein stability changes accurately. In this case, the classification task is a good choice for predicting stability changes. Dividing the mutations into destabilizing and stabilizing mutations, the binary cross-entropy loss function measures the difference between predicted probabilities and actual binary labels. With limited training data, the classification task can let the model predict if the mutations can increase the stability of the proteins. Therefore, we will apply classification metrics, such as accuracy, recall, precision, and AUROC.

**2. An overview of the GCN method**

The pre-processing procedure for the GCN-based method is the same as ProstaNet. First, the 3D structures of wild-type and mutant proteins are converted into two structural graphs. The protein sequences are embedded using amino acid encoding methods and used as the node features within the graphs. An adjacency matrix is used as the edge feature. These graphs are then passed through two GCN layers to extract features separately. A global pooling layer then is added after each of the GCN layers to perform max operation on the GCN layers, which fixes the size of representations for all proteins. Following the pooling layer, a dense layer with a ReLU activation function is added to get the final representation of a protein, outputting a feature vector. The two feature vectors from the two graphs are concatenated and passed through dense layers to make the classification prediction

**3. The overview of data augmentation methods**

The amount of data, especially for multiple-point mutations, is insufficient, which has affected the learning ability of the model. Therefore, we need to augment the data. Because of amino acids properties and close relationship between protein structure and its stability, some mutation types have a lower rate appear in both single-point and multiple-point mutations (**Supplementary Figure 1**). Our predictor is for researchers to predict if the mutation they want to perform increase protein stability. Hence, the distribution of data mutation types needs to be similar to the real-world situation. A state-of-the-art data augmentation technique thermodynamic permutation^1^ (TP), which made distribution of all mutation types become balanced through state-function property of Gibbs free energy. The balanced distribution of mutation types will let the model move the time from learning the pattern of common mutation types to the pattern of some rare mutation types, which affect the performance of model in predicting the data under the real-world situation. Meanwhile, the calculation of ΔΔG value is not just the numerical addition or subtraction. An excellent ΔΔG predictor should be “self-consistent”, accurately predicting ΔΔG values for both direct and reverse mutations. However, previous ΔΔG predictors have often overlooked this requirement^2, 3^. Therefore, TP is not a suitable method in our case.

**4. Thermodynamic data collection**

Because most of the thermodynamic data in MPTherm and ProThermDB databases is shown in ΔT_m_ value. So, we collected data that has ΔT_m_ or ΔΔG values. We collected data with ΔΔG value in ThermoMutDB, FireProt, S2648, and S^sym^ databases. We identified data whose ΔT_m_ larger than 0 ^o^C and ΔΔG smaller than 0 kcal/mol as the stabilizing mutation, otherwise known as destabilizing mutation.

**5. Thermodynamic data augmentation methods**

The thermodynamic looping method is based on the concept of conservation of energy and state function. Gibbs free energy value depends only on the initial and final states. If a system is a complete cycle, where the system returns to its original state, the total ∆∆G changes must be zero. If both variant A and variant B share the same wild-type. Based on experimental data, variant A has a higher energy state and variant B has a lower energy state compared with their wild-type. Wild-type mutates to variant A with ΔΔG > 0 kcal/mol, variant B mutates back to wild-type with ΔΔG >0 kcal/mol. To ensure energy conservation between circle of wild-type, variant A, and variant B, the ΔΔG value of variant A mutates to variant B should be lower than 0 kcal/mol. We used protein tyrosine kinase (PDB: 1a0n) as an example. Wild-type 1a0n has amino acids alanine and valine on position 132 and 148 respectively. The double point mutation A132V, V148I has been proved by the experiment that with ∆∆G lower than 0 kcal/mol, which means that the energy of A132V, V148I mutant is lower than its wild-type. Another double point mutation A132G, V148F for 1a0n has been proved by the experiment that with ∆∆G larger than 0 kcal/mol. According to thermodynamic reversibility, if the mutation is from G132, F148 to its wild-type, the ∆∆G should be the negative of ∆∆G_A132G,V148F._ Therefore, to make the sum of ∆∆G of the total system to be zero, we can make the hypothesis that the ∆∆G of mutation from V132, I148 to G132, F148 is larger than 0 kcal/mol.

Thermodynamic reversibility augmentation method is based on the concept of thermodynamic equilibrium. Two proteins that are nearly identical in sequence, differing only at one specific position where one has the amino acid X while the other has Y. We defined protein with amino acid X as protein X and with amino acid Y as protein Y. Let their gibbs free energy of folding as ΔG_X_ and ΔG_Y_ respectively. The protein Y is generated from amino acid X mutating to Y was called a direct mutation and the change of folding Gibbs free energy of two proteins:

${\Delta\Delta G}_{X\to Y} = {\Delta G}_{Y} - {\Delta G}_{X}$ (1)

On the contrary, the protein X as being generated from amino acid Y to X is called a reverse mutation and its folding Gibbs free energy:

${\Delta\Delta G}_{Y\to X}= {\Delta G}_{X}- {\Delta G}_{Y}= -{\Delta\Delta G}_{X\to Y}$ (2)

**6. Protein thermodynamic stability changes upon mutations**

The Gibbs free energy for the process of protein folding is represented by the thermodynamic stability of a protein (ΔG). The ΔG of a protein can be measured by the difference between the Gibbs free energy of unfolded and folded states

$\Delta G=G_{\left( unfolded \right)}-G_{\left( folded \right)}$ (1)

The folded state achieves lower Gibbs free energy than the unfolded state. Therefore, the higher ΔG, the more stable of the protein folded state. The ΔG is influenced by the interactions occurring among amino acid residues within the protein^4^. When the amino acids substitution occurs in a protein, it may affect the interaction of amino acids within the protein and lead to thermodynamic stability changes. The protein without mutation is called wild-type and its Gibbs free energy of folding is denoted as ΔG_(wildtype)_. On the contrary, the protein with amino acid mutation is called mutant and its Gibbs free energy of folding is referred to as ΔG_(mutant)_. Hence, when the protein stability changes, ΔΔG can be obtained from the difference between Gibbs free energy of folding of wild-type and mutant proteins^5^:

${\Delta\Delta G}_{dir}= {\Delta G}_{\left( mutant \right)}- {\Delta G}_{\left( wildtype \right)}$ (2)

With equation (1) and equation (2), negative values of ΔΔG_dir_ indicates destabilizing mutations, while positive ΔΔG_dir_ value indicates stabilizing mutations.

**7. Graph networks**

GNN has been developed, which can process protein structure graphs and has been proven to have good performance in protein property predictions. With the rapid development in GNNs, it has derived several variants. Here we describe two GNN variants GVP-GNN and GCN that we used in our work. For both GVP-GNN and GCN, the protein 3D structure was transformed into a graph $\mathcal{G} = \left( \mathcal{V,E} \right)$. The amino acid on the given protein was represented by the node $v_{\mathcal{i}} \text{∈ }\mathcal{V}$ and had node features $h_{v}^{\left( i \right)}$. The connection between amino acids was represented by the edges in the graph and had edge features $h_{e}^{(j\to i)}$.

**GVP-GNN layer.** The node $h_{v}^{(i)}=\left( s_{v}^{\left( i \right)}, V_{v}^{\left( i \right)} \right) \in\mathbb{R}^{n} \times\mathbb{R}^{v \times3}$ and edge $h_{e}^{(j\to i)}=\left( s_{e}^{\left( j\to i \right)}, V_{e}^{\left( j\to i \right)} \right) \in\mathbb{R}^{m} \times\mathbb{R}^{\mu\times3}$ features in the protein graph process to GVP-GNN are composed of scalar and vector features. During GVP-GNN graph propagation, node embeddings are updated at each step by leveraging message passing, where messages from neighboring nodes and edges are utilized. GVP-GNN takes the protein graph and performs graph propagation according to:

$h_{\mathcal{m}}^{(j\to i)} :=g\left( concat\left( h_{\nu}^{\left( j \right)}, h_{e}^{\left( j\to i \right)} \right) \right)$ (3)

$h_{v}^{(i)} \leftarrow LayerNorm (h_{v}^{\left( i \right)}+\frac{1}{k^{'}}\mathrm{Dropout}(\sum h_{m}^{\left( j\to i \right)}))$ (4)

In equation (5) and equation (6), g represents a sequence of several GVPs and $h_{v}^{(j)}$ is the message passed from node $j$ to node $i$. $k^{'}$ represents the number of incoming messages, which is equal to the number of residues on the given sequences. Between steps of graph propagation, a feed-forward point-wise layer is used to update the node embeddings across all nodes $i$.

$h_{v}^{(i)} \leftarrow\mathrm{LayerNorm}\left( h_{v}^{\left( i \right)}+\mathrm{Dropout}\left( g\left( h_{v}^{\left( i \right)} \right) \right) \right)$ (5)

In equation (7), g represents a sequence of two GVPs. The graph propagation and feed-forward steps serve to update both the vector and scalar features at each node.

**GCN layer.** Except for nodes and edges, the graph process to the GCN layer with adjacency matrix $A \in\mathbb{R}^{N \times N}$ and a degree matrix $D_{ii}= \sum_{j} A_{ij}$. Unlike the nodes in the graph process to GVP-GNN layer, the nodes in the graph process to GCN layer just contain scalar features. Every neural network layer in GCN takes the node embedding from the previous layer that can be written as:

$h^{(l+1)}=f(h^{\left( l \right)}, A)$ (6)

Where $l$ represents the number of layers. $h^{(l)}$ is the node embedding of layer $l$. GCN takes the protein graph and performs graph propagation according to:

$h^{(l+1)} =activation\left( \hat{D}^{-0.5}\hat{A}\hat{D}^{-0.5}h^{\left( l \right)}W^{\left( l \right)} \right)$ (7)

In equation (9), $W^{(l)}$is a layer-specific trainable weight matrix and $\hat{D}$ is the diagonal node degree matrix. $\hat{A}=A+I_{N}$ is the adjacency matrix of the graph with added self-connections, $I_{N}$ is the identity matrix. $(\hat{D}^{-0.5}\hat{A}\hat{D}^{-0.5})$is used to normalize the adjacency matrix to get the normalized residue features after each layer, and each features vector is updated by weighted sum of its own feature and the features of neighboring nodes in the graph.

**8.** **Representation of proteins**

A protein structure was represented by a proximity graph with a scalar and vector features to specify the 3D structure of the molecule. $i \text{∈}$ 1, 2, 3, …, *L* designate as the position of amino acid in the protein sequence of length *L*. The protein structure was represented as a graph $\mathcal{G} = \left( \mathcal{V,E} \right)$ following Ingraham *et al*.^6^ and Jing^7^. An amino acid is represented by each node $v_{i} \text{∈ }\mathcal{V}$ in the graph and has node features $h_{v}^{\left( i \right)}$ with scalar $s_{\nu}^{\left( i \right)}$ and vector $V_{\nu}^{(i)}$ features, scalar features were generated by the following encoding methods:

- 20 dimension (20D) representation from the one-hot encoding method^8^. The twenty standard amino acids were arranged in a predetermined order, and then the amino acid type in $i$th position is represented by twenty binary bits with the $i$th bit set to “1” and others to “0”.
- 5D represented twenty standard amino acids based on their attributes in five properties: polarity, molecular volume, codon diversity, secondary structure, and electrostatic charge^9^ (Acthely factors).
- 20D represented the log-likelihoods of the occurrence probabilities of twenty amino acids at each location in a given sequence. The sequences of all the data in ProstaDB and S^sym^ were used to perform multiple sequence alignments against sequences in Uniref90 database by using Position-Specific Iterative Basic Local Alignment Search Tool (PSI-BLAST)^10^, with e-value 0.001.
- 20D represented optimal interaction potentials between the amino acids^11^ (Micheletti potentials).
- 20D representation from Rosetta scoring functions, including both knowledge-based and physics-based energy terms^12^, which were extracted from the relaxed and mutated protein 3D structures after applying Rosetta application.
- 6D representation from dihedral angles $\varphi, \omega, \phi$ computed from $C_{i-1}$, $N_{i}$, $N_{i+1}$, $C_{\alpha_{i}}$, and $C_{i}$^45^.
- 1024D representation from ProtBert^13^, which trained on the BFD-100 dataset^14, 15, 16^ and employed the BERT model^17^ to generate descriptive features for each residue in the protein sequences.

Vector features were included forward and reverse unit vectors in the directions of $C_{\alpha_{i}}$ to its two neighboring amino acids; the unit vector in the inputed direction of $C_{\beta_{i}}$ to $C_{\alpha_{i}}$. These vectors determine the orientation of each amino acid.

The set of edges is $\mathcal{E =}\left( e_{j\to i} \right)$, $i, j$ were the positions of amino acids on given protein sequence. Measured by the distance between $C_{\alpha}$ atoms, the K = 30 nearest neighbors of $v_{i}$ was defined as $v_{j}$. Same as node features, edge features$h_{e}^{(j\to i)}$ were composed of scalar $s_{e}^{(j\to i)}$ and vector $V_{e}^{(j\to i)}$ features. Scalar features encompass the representation of $C_{\alpha}$ distances using 16 Gaussian radial basis functions, evenly distributed across the range of 0 to 20$Å$; the encoding of $j - i$ distance along the amino acids backbone as described in Vaswani *et al.*^18^. The vector features included the unit vector in the direction of $C_{\alpha}$atom between $i\mathrm{th}, j\mathrm{th}$ position.

**9. ProstaDB data record**

The ProstaDB database consists of two parts. The first part is an excel spreadsheet (ProstaDB.xlsx) that includes both single-point and multiple-point mutations data and is available on <https://github.com/NikoBelice/ProstaNet>. The second part is a zip file containing the PDB files of all structures in the ProstaDB database, which is available on <https://doi.org/10.5281/zenodo.14658434>. ProstaDB.xlsx consists of two sheets, single-point mutation and multiple-point mutation.

**Data format of single-point mutation**

(1) PDB_wild_type: the name of wild_type protein PDB file

(2) PDB: PDB code and mutation chain of protein, [PDB code] + [chain ID]

(3) Position: the position of the mutated amino acid

(4) Parent_amino_acid

(5) Mutant_amino_acid

(6) PDB_mutant: the name of mutant protein PDB file, [PDB] + [Mutation_combination]

(7) Stability_changes: the thermostability changes of mutation, 1 indicates stabilizing mutation, 0 indicates destabilizing mutation

**Data format of multiple-point mutation**

(1) PDB_wild_type: the name of wild_type protein PDB file

(2) PDB: PDB code and mutation chain of protein, [PDB code] + [chain ID]

(3) Mutation_combination: information of multiple-point mutation, [parent amino acid] + [position] + [mutated amino acid]

(4) PDB_mutant: the name of mutant protein PDB file, [PDB] + [Mutation_combination]

(5) Stability_changes: the thermostability changes of mutation, 1 indicates stabilizing mutation, 0 indicates destabilizing mutation


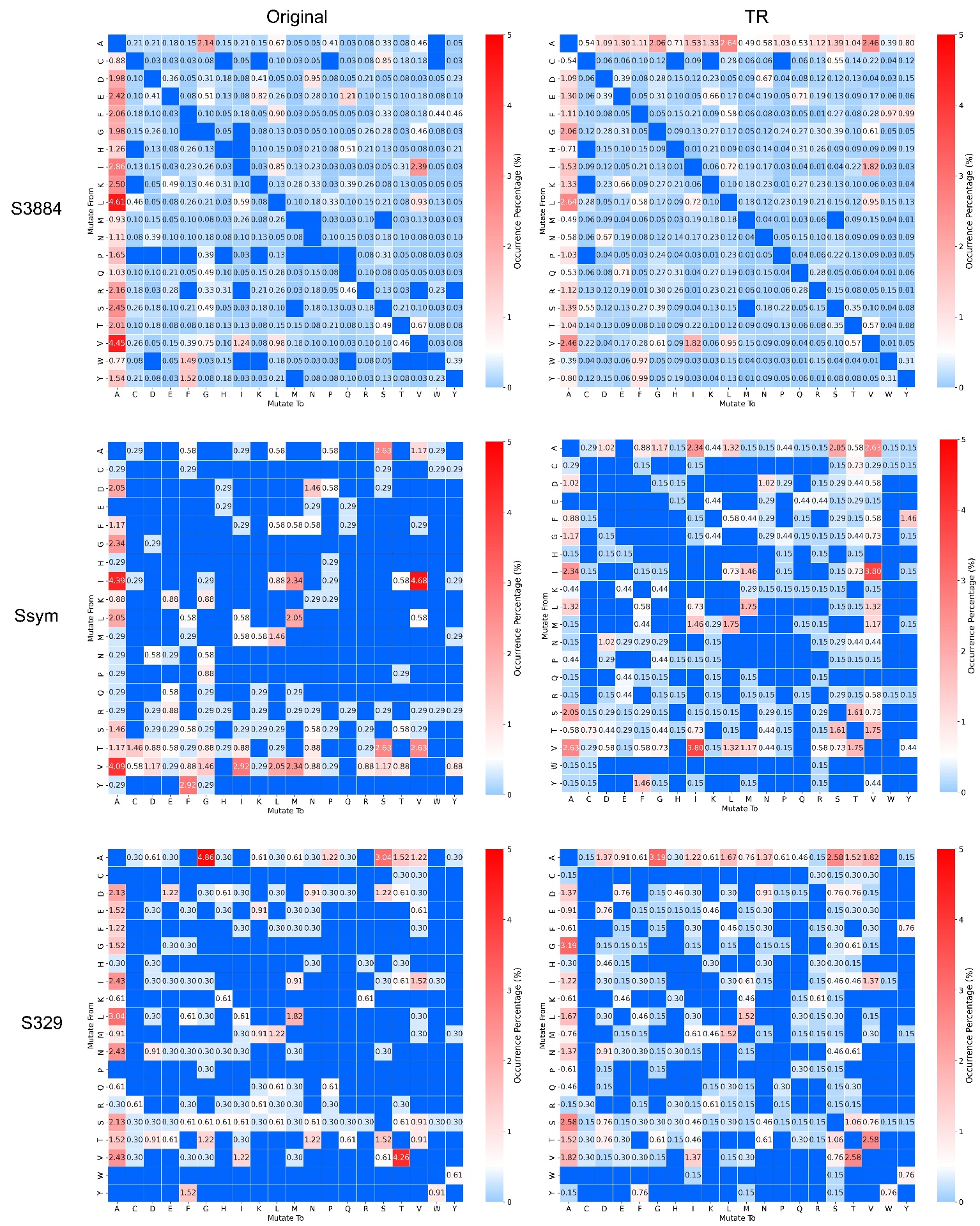


**Supplementary Figure 1 | The mutation type distribution of single-point mutation datasets by using different data augmentation methods.** Heatmap representation mutation type distribution in single-point mutation datasets. The cell in dark blue represents this mutation type does not occur in this dataset. Mutation type distribution in original and TR augmentation S3885, S^sym^, and S329 datasets.

a


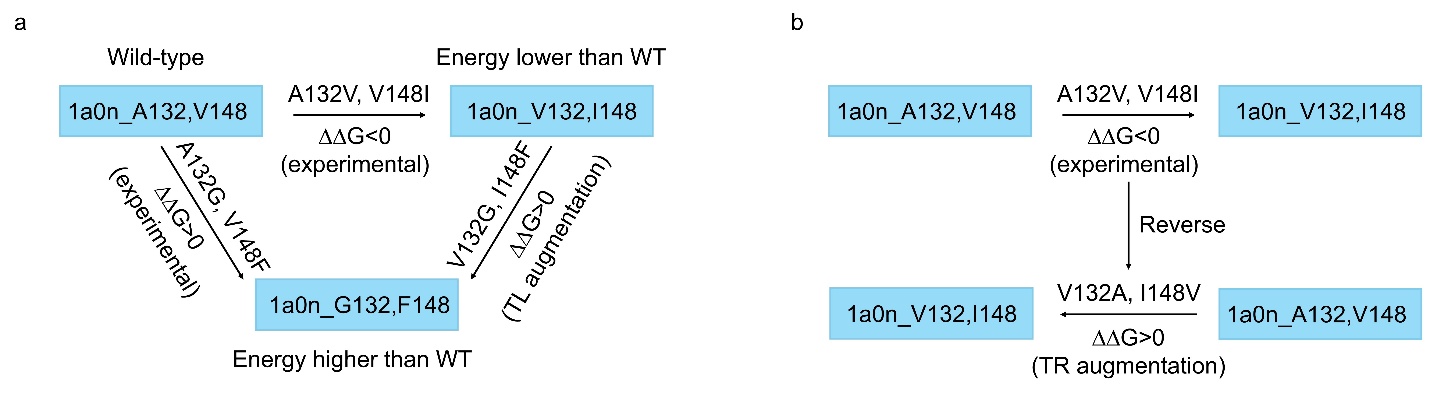


**Supplementary Figure 2 | Thermodynamic looping and thermodynamic reversibility.** Thermodynamic data augmentation methods that were applied in the datasets. a. The mechanism of how thermodynamic looping works. b. The mechanism of how thermodynamic reversibility works.


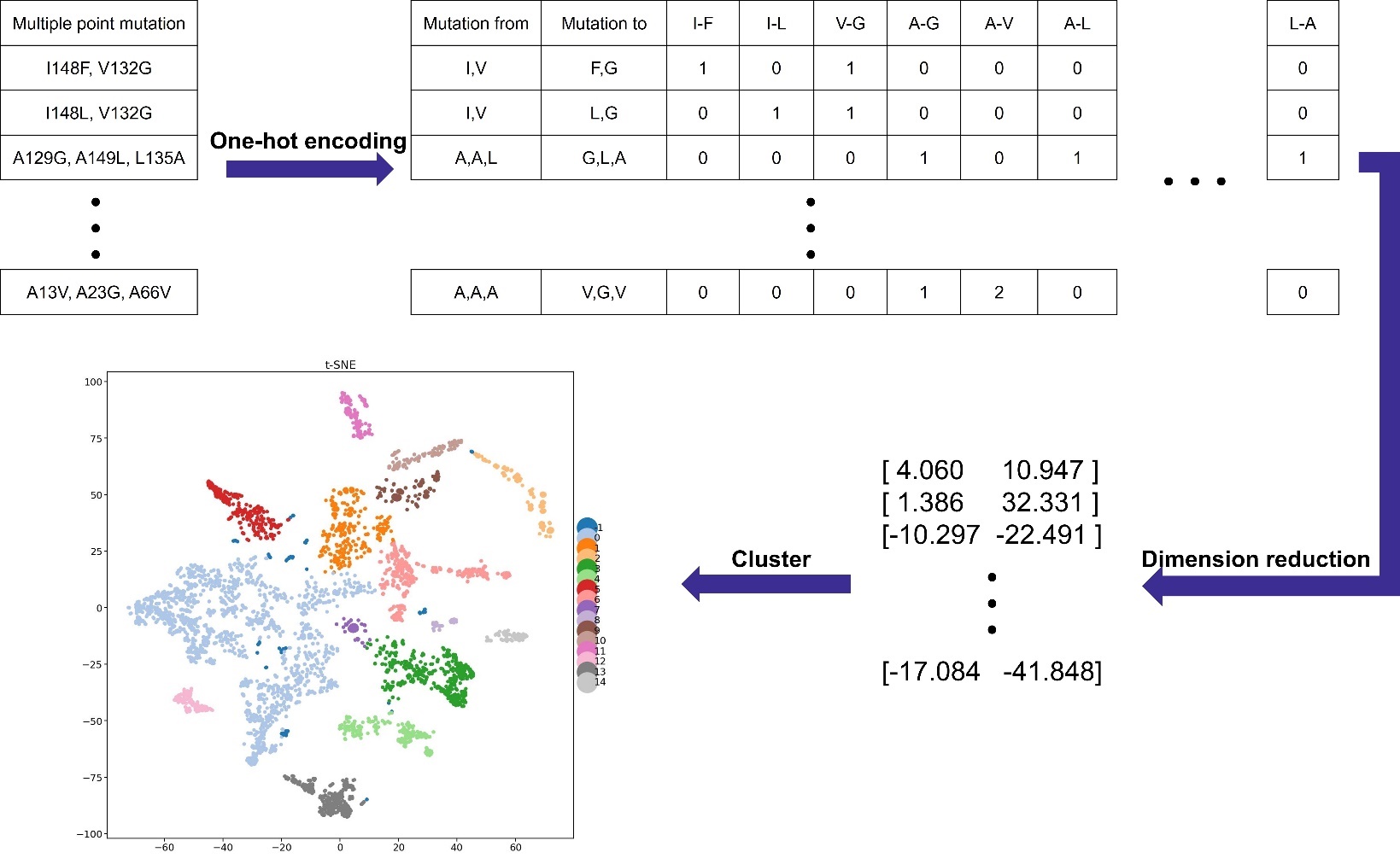


**Supplementary Figure 3 | The procedure of clustering method that used to gain the testing set.** The multiple-point mutation is considered as the combination of different mutation types. A-B is a mutation type, which means the mutation is from amino acid A to amino acid B. If a multiple-point mutation contains certain mutation types, 1’ will be marked under those mutation types. Different colors in DBSCAN result plot represent different clusters.


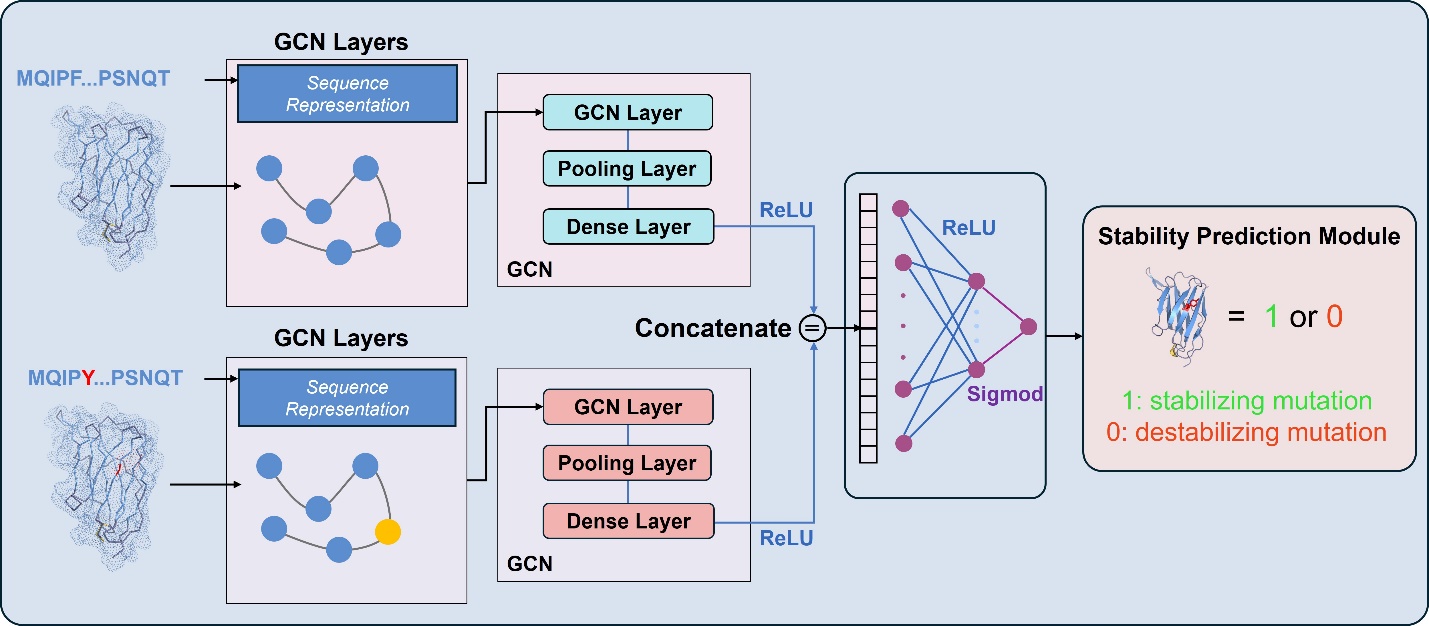


**Supplementary Figure 4 | The architecture of GCN-based method.** The 3D structures preprocessing process of GCN-based method is same as ProstaNet. The sequences of wild-type and mutant proteins are embedded and served as scalar vector of the nodes. Structure graphs are passed to GCN layers, followed by a pooling layer and a dense layer. The features vector of wild-type and mutant are concatenated, then pass to the dense layers and output “0” or “1”.


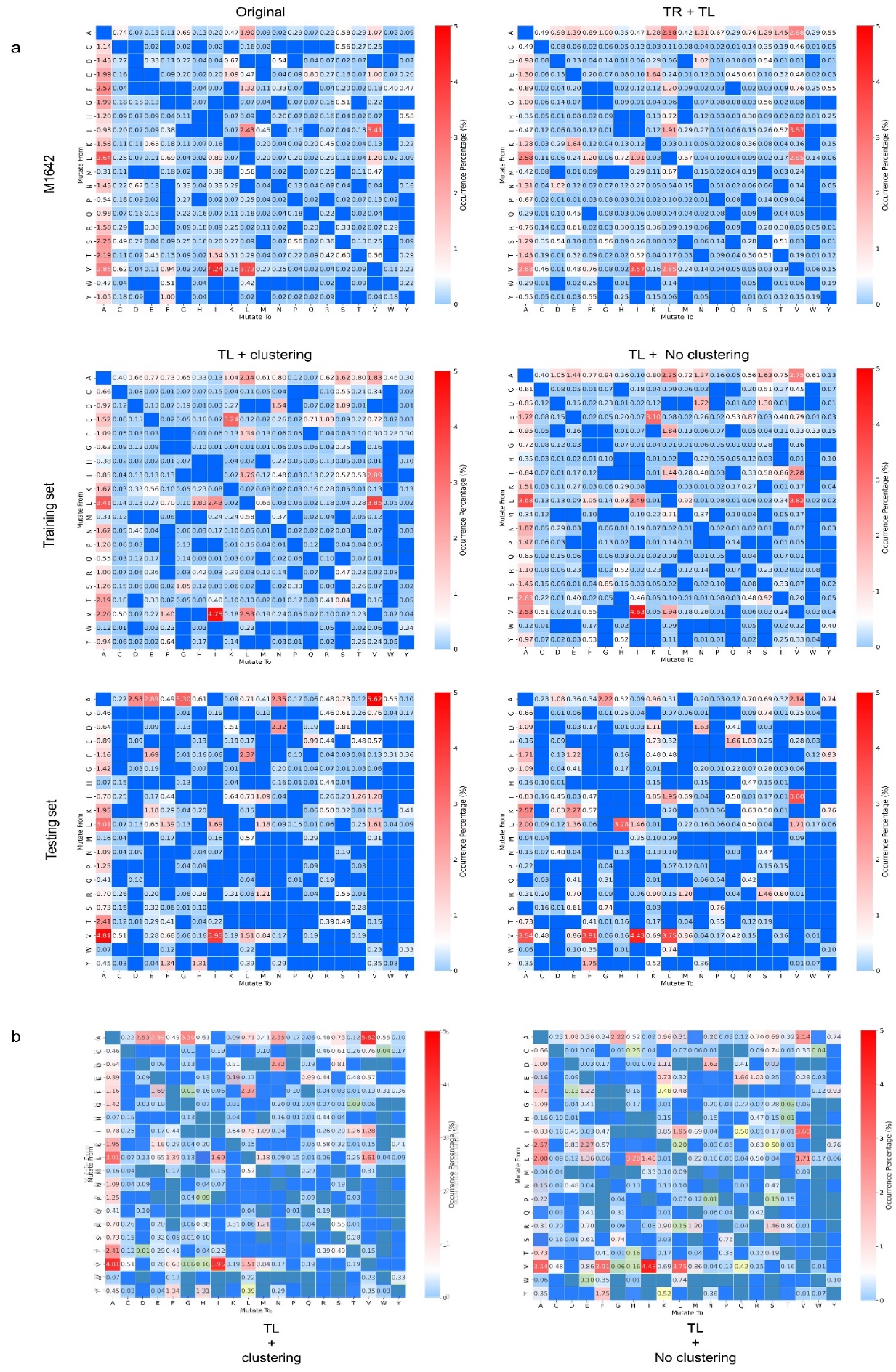


**Supplementary Figure 5 | The mutation type distribution of different datasets by using different data augmentation methods. a.** Mutation type distribution of original and TL+TR M1642 datasets. Mutation type distribution of training and testing sets split from TL+M1642 with or without using clustering method. **b.** Overlap heatmaps of training and testing sets split from TL+M1642 with or without using clustering method. The yellow cell with number represents the mutation type only occurs in testing set.

**Table S1. The characteristics of state-of-the-art methods.**

| **Method** | **Algorithm** | **Features** |
| --- | --- | --- |
| **Structure-based** | | |
| ThermoNet | 3D-convolutional neural network | Multi-channel voxel grids based on biophysical properties |
| ThermoMPNN | Message-passing neural network | Pairwise atomic distances, sequence information from large language models |
| PoPMuSiC^sym^ | Linear parametric with coefficients depend on a neural network | 13 Statistical potentials, 2 amino acids volume |
| FoldX | NA | Empirical force field |
| DDGun3D | Linear parametric model | Evolutionary information, substitution matrix, interaction energy, and hydrophobicity |
| MAESTRO | Combination of multiple of machine learning methods | Statistical scoring functions |
| **Sequence-based** | | |
| ACDC-NN-Seq | Neural networks methods | One-hot encoding, profile information of amino acids and its neighborhood |
| MUPRO | SVM-based methods | Sequence and structure information |

**Table S2. The detailed description for removing duplicate and homology data within the datasets.**

| **Process** | **Detailed description** |
| --- | --- |
| Remove duplication | Duplicate data are removed from the dataset by ensuring that each mutation, defined by the same wild-type protein (same PDB number) and identical mutation, appears only once across databases. This guarantees that the dataset does not contain redundant mutation data. All newly collected data will be compared with the existing dataset. If a mutation is found to be a duplicate of previously collected data, it will be excluded. |
| Combine dataset | After removing duplicate data, all the single-point or multiple-point mutation data collected from different databases will be combined to form single-point and multiple point mutation datasets, respectively. |
| Remove homologous data | BLASTp is used to identify homologous proteins within the dataset, with the BLAST e-value threshold set to less than 0.001. BLASTp is applied to all wild-type proteins in the dataset and outputs other proteins within the dataset that share significant sequence homology with them. If two wild-type proteins are homologous, the one with fewer mutations will be removed. |

**Table S3. The clusters of the multiple-point mutation data.**

| **Cluster number** | **Data number in each cluster** | **Data number in testing set** |
| --- | --- | --- |
| -1 | 53 | 7 |
| 0 | 1188 | 236 |
| 1 | 2663 | 561 |
| 2 | 73 | 9 |
| 3 | 771 | 155 |
| 4 | 182 | 36 |
| 5 | 624 | 122 |
| 6 | 457 | 90 |
| 7 | 462 | 91 |
| 8 | 142 | 29 |
| 9 | 42 | 20 |
| 10 | 124 | 12 |
| 11 | 133 | 6 |
| 12 | 210 | 0 |
| 13 | 135 | 31 |
| 14 | 101 | 18 |

**Table S4. The requirements and runtime of each method.**

| **Method** | **Requirements** | **Runtime**  **(Efficiency)** |
| --- | --- | --- |
| ProstaNet | No mutation list required  All essential codes are provided, just run the provided codes | 0.0047s/structure |
| ThermoMPNN | No detailed instructions  Users need to modify the codes and set up their custom dataset | 5.41s/structure |
| FoldX | A mutation list needs to be prepared in a specific format | 0.37s/structure |
| MAESTRO | A mutation list needs to be prepared in a specific format | 0.12s/structure |
| MUPRO | A running file needs to be prepared in a specific format | 0.03s/structure |
| DDGun3D | A mutation list needs to be prepared  An additional 80GB database is required  Multiple sequence alignment (MSA) must be performed before prediction | 26s/structure |
| ACDC-MM-Seq | MSA is required before prediction  The running time of MSA is longer | 2s/structure |

**Table S5. The direct and reverse mutations accuracy of different models in protein thermostability prediction upon S^sym^ testing set.**

|  | **Direct_mutation** | **Reverse_mutation** |
| --- | --- | --- |
| Full | 0.75 | 0.74 |
| w/o Binary | 0.75 | 0.74 |
| w/o Physicochemical properties | 0.75 | 0.68 |
| w/o Evolution-based | 0.71 | 0.71 |
| w/o Structure-based | 0.76 | 0.74 |
| w/o Residue scoring | 0.58 | 0.61 |
| w/o Conformation properties | 0.76 | 0.75 |
| GCN_ProtBert | 0.57 | 0.57 |
| GCN_Full | 0.75 | 0.70 |
| S7768_finetune | 0.75 | 0.74 |
| S7768+M11872_train | 0.77 | 0.76 |
| S3884  (Without TR and TL augmentation) | 0.75 | 0.26 |

**Table S6. The four metrics of different models in protein thermostability prediction upon S^sym^ testing set.**

|  | **Accuracy** | **Precision** | **Recall** | **AUC** |
| --- | --- | --- | --- | --- |
| Full | 0.75 | 0.74 | 0.75 | 0.73 |
| w/o Binary | 0.75 | 0.74 | 0.75 | 0.73 |
| w/o Physicochemical properties | 0.72 | 0.76 | 0.64 | 0.76 |
| w/o Evolution-based | 0.75 | 0.74 | 0.75 | 0.72 |
| w/o Structure-based | 0.75 | 0.74 | 0.75 | 0.72 |
| w/o Residue scoring | 0.59 | 0.59 | 0.64 | 0.65 |
| w/o Conformation properties | 0.75 | 0.74 | 0.75 | 0.74 |
| GCN_ProtBert | 0.57 | 0.57 | 0.59 | 0.59 |
| GCN_Full | 0.72 | 0.73 | 0.69 | 0.73 |
| S7768_finetune | 0.75 | 0.74 | 0.75 | 0.74 |
| S7768+M11872_train | 0.76 | 0.74 | 0.79 | 0.82 |

**Table S7. The direct and reverse mutations accuracy of different models in protein thermostability prediction upon M2848 testing set.**

|  | **Direct_mutation** | **Reverse_mutation** |
| --- | --- | --- |
| Full | 0.94 | 0.92 |
| w/o Binary | 0.93 | 0.91 |
| w/o Physicochemical properties | 0.93 | 0.92 |
| w/o Evolution-based | 0.77 | 0.75 |
| w/o Structure-based | 0.89 | 0.93 |
| w/o Residue scoring | 0.86 | 0.82 |
| w/o Conformation properties | 0.92 | 0.89 |
| GCN_ProtBert | 0.75 | 0.72 |
| GCN_Full | 0.54 | 0.65 |
| M11872_pretrain | 0.82 | 0.83 |
| S7768+M11872_train | 0.82 | 0.80 |
| M1642+TR+no_cluster  (Without TL augmentation) | 0.60 | 0.53 |
| M1642 + no_cluster  (Without TL + TR augmentation) | 0.59 | 0.43 |
| M1642+TL+TR+no_cluster  (With augmentation) | 0.67 | 0.62 |

**Table S8. The four metrics of different models in protein thermostability prediction upon M2848 testing set.**

|  | **Accuracy** | **Precision** | **Recall** | **AUC** |
| --- | --- | --- | --- | --- |
| Full | 0.93 | 0.96 | 0.90 | 0.99 |
| w/o Binary | 0.92 | 0.93 | 0.92 | 0.98 |
| w/o Physicochemical properties | 0.92 | 0.93 | 0.92 | 0.98 |
| w/o Evolution-based | 0.76 | 0.76 | 0.76 | 0.87 |
| w/o Structure-based | 0.91 | 0.88 | 0.95 | 0.98 |
| w/o Residue scoring | 0.84 | 0.86 | 0.81 | 0.94 |
| w/o Conformation properties | 0.91 | 0.94 | 0.87 | 0.98 |
| GCN_ProtBert | 0.78 | 0.79 | 0.76 | 0.87 |
| GCN_Full | 0.59 | 0.58 | 0.67 | 0.63 |
| M11872_pretrain | 0.83 | 0.84 | 0.81 | 0.93 |
| S7768+M11872_train | 0.81 | 0.82 | 0.79 | 0.92 |
| M1642+TR+no_cluster | 0.57 | 0.56 | 0.62 | 0.58 |
| M1642+TL+TR+no_cluster | 0.65 | 0.66 | 0.63 | 0.75 |

**References**

1. Diaz DJ*, et al.* Stability Oracle: a structure-based graph-transformer framework for identifying stabilizing mutations. *Nature Communications* **15**, 6170 (2024).

2. Thiltgen G, Goldstein RA. Assessing Predictors of Changes in Protein Stability upon Mutation Using Self-Consistency. *PLOS ONE* **7**, e46084 (2012).

3. Usmanova DR*, et al.* Self-consistency test reveals systematic bias in programs for prediction change of stability upon mutation. *Bioinformatics* **34**, 3653-3658 (2018).

4. Li B, Fooksa M, Heinze S, Meiler J. Finding the needle in the haystack: towards solving the protein-folding problem computationally. *Critical Reviews in Biochemistry and Molecular Biology* **53**, 1-28 (2018).

5. Gong H*, et al.* Unbiased curriculum learning enhanced global-local graph neural network for protein thermodynamic stability prediction. *Bioinformatics* **39**, btad589 (2023).

6. Ingraham J, Garg VK, Barzilay R, Jaakkola T. Generative Models for Graph-Based Protein Design. In: *DGS@ICLR*) (2019).

7. Jing B, Eismann S, Suriana P, Townshend RJL, Dror R. Learning from protein structure with geometric vector perceptrons. In: *International Conference on Learning Representations*) (2020).

8. Cheng J, Tegge AN, Baldi P. Machine Learning Methods for Protein Structure Prediction. *IEEE Reviews in Biomedical Engineering* **1**, 41-49 (2008).

9. Atchley WR, Zhao J, Fernandes AD, Drüke T. Solving the protein sequence metric problem. *Proceedings of the National Academy of Sciences* **102**, 6395-6400 (2005).

10. Altschul SF, Koonin EV. Iterated profile searches with PSI-BLAST--a tool for discovery in protein databases. *Trends Biochem Sci* **23**, 444-447 (1998).

11. Micheletti C, Seno F, Banavar JR, Maritan A. Learning effective amino acid interactions through iterative stochastic techniques. *Proteins* **42**, 422-431 (2001).

12. Alford RF*, et al.* The Rosetta All-Atom Energy Function for Macromolecular Modeling and Design. *Journal of Chemical Theory and Computation* **13**, 3031-3048 (2017).

13. Ahmed E*, et al.* ProtTrans: Towards Cracking the Language of Life’s Code Through Self-Supervised Learning. *bioRxiv*, 2020.2007.2012.199554 (2021).

14. Jumper J*, et al.* Highly accurate protein structure prediction with AlphaFold. *Nature* **596**, 583-589 (2021).

15. Steinegger M, Mirdita M, Söding J. Protein-level assembly increases protein sequence recovery from metagenomic samples manyfold. *Nature Methods* **16**, 603-606 (2019).

16. Steinegger M, Söding J. Clustering huge protein sequence sets in linear time. *Nature Communications* **9**, 2542 (2018).

17. Devlin J, Chang M-W, Lee K, Toutanova K. Bert: Pre-training of deep bidirectional transformers for language understanding. *arXiv preprint arXiv:181004805*, (2018).

18. Vaswani A*, et al.* Attention is all you need. *Advances in neural information processing systems* **30**, (2017).
