# Supplementary figures and images for: ProstaNet: A Novel Geometric Vector Perceptrons–Graph Neural Network Algorithm for Protein Stability Prediction in Single- and Multiple-Point Mutations with Experimental Validation
Source: Research (Wash D C). 2025 Apr 15;8:0674. doi: 10.34133/research.0674 (PMC11997553; doi:10.34133/research.0674)

Original

TR

S3884

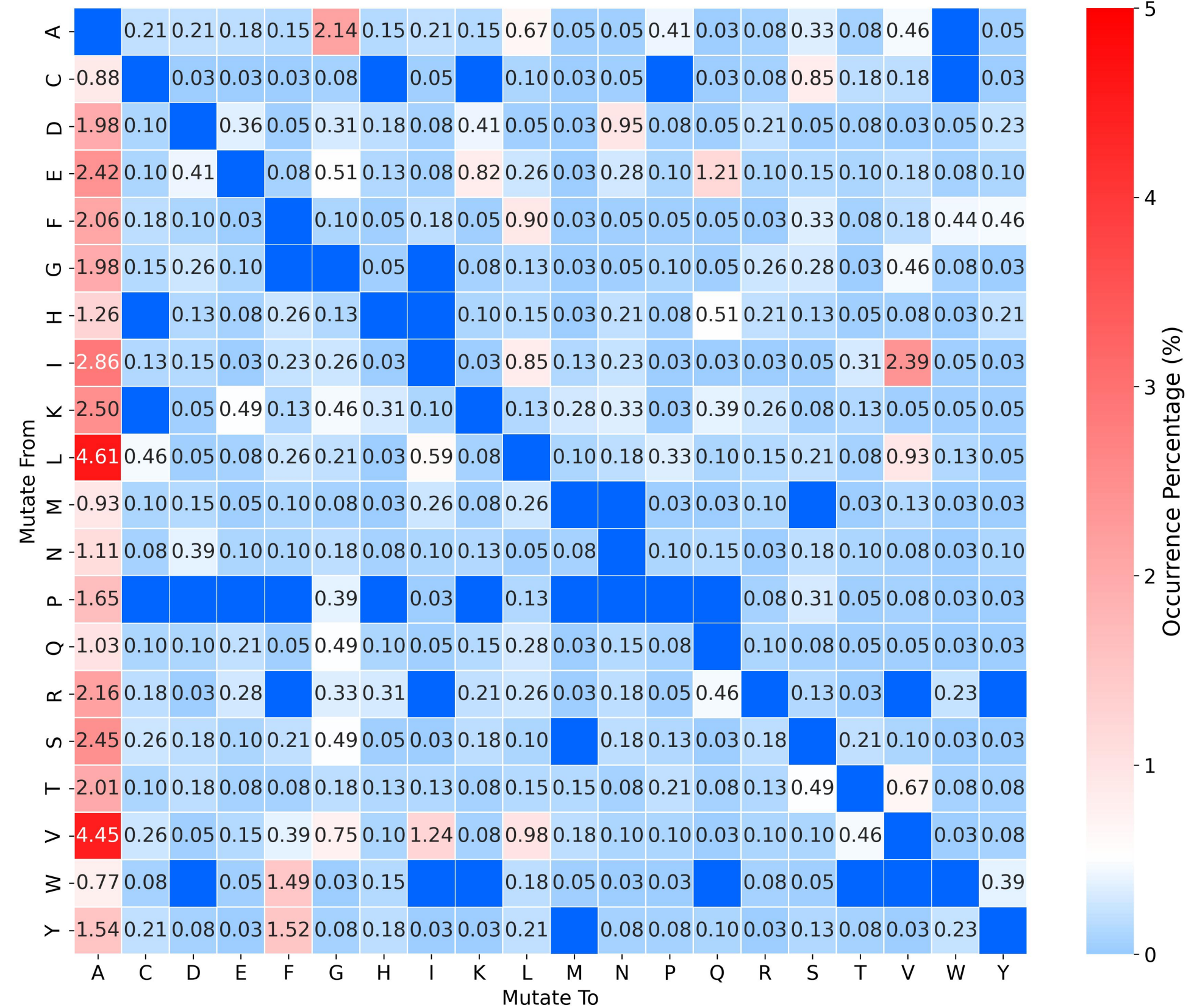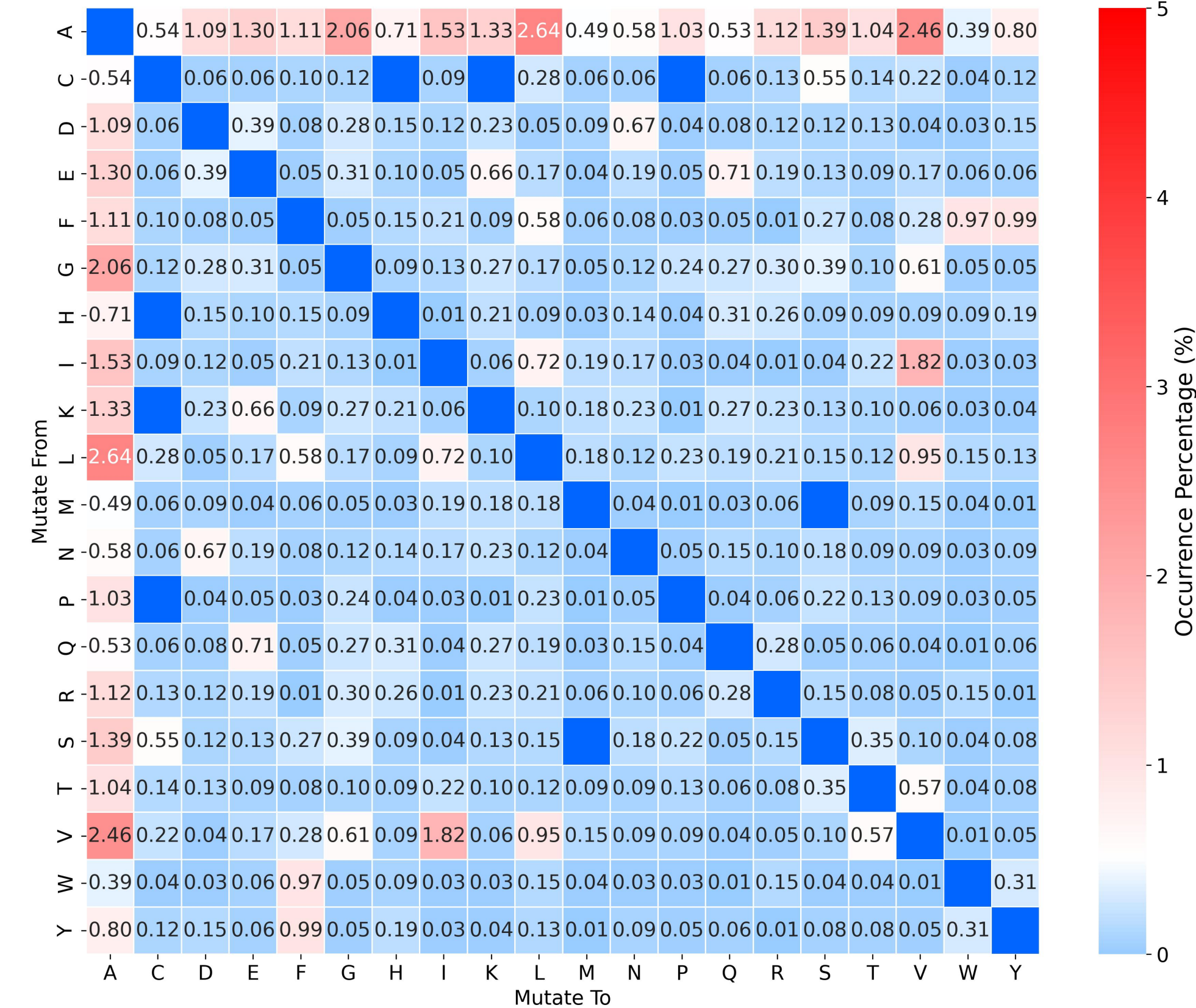

Ssym

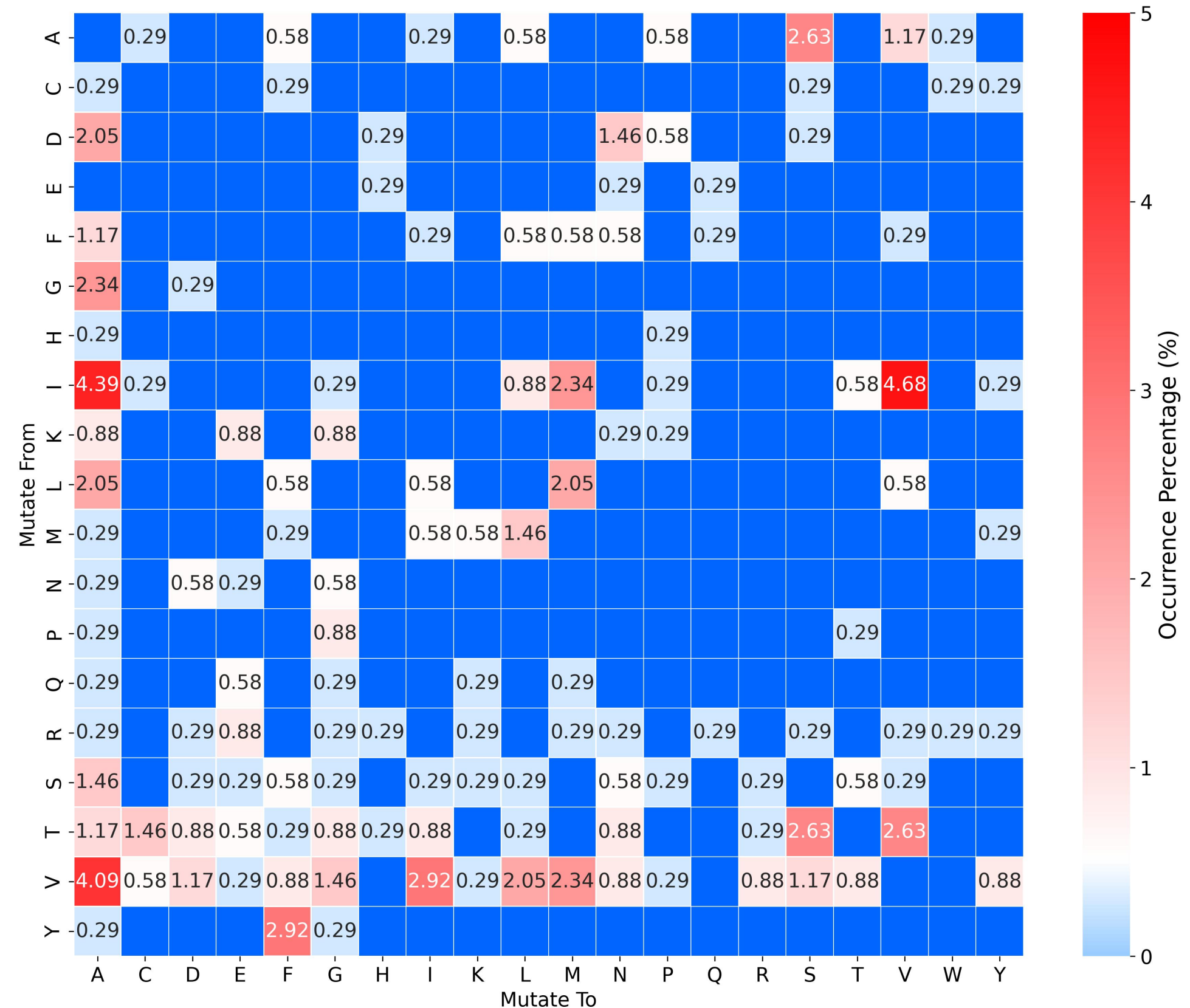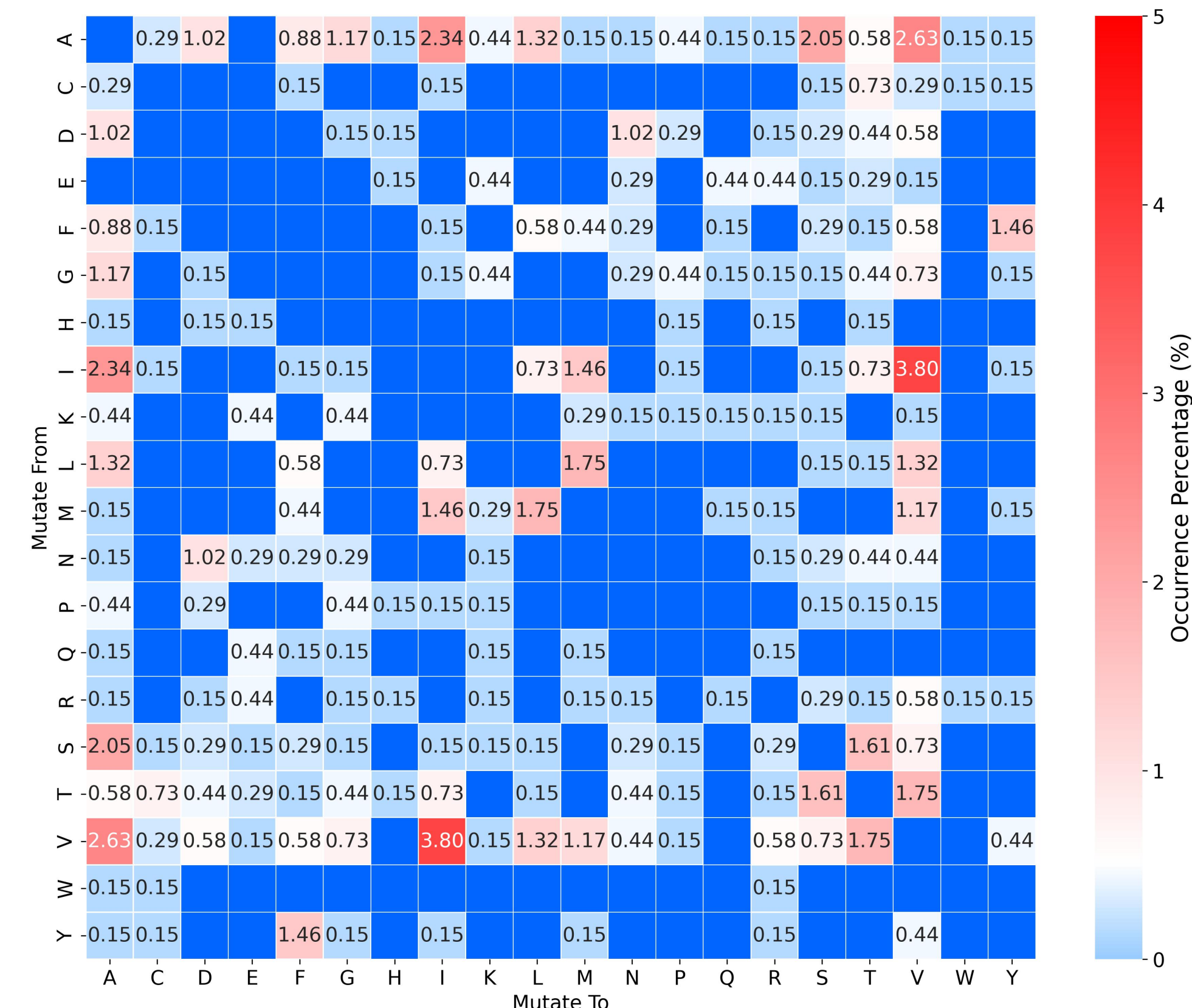

S329

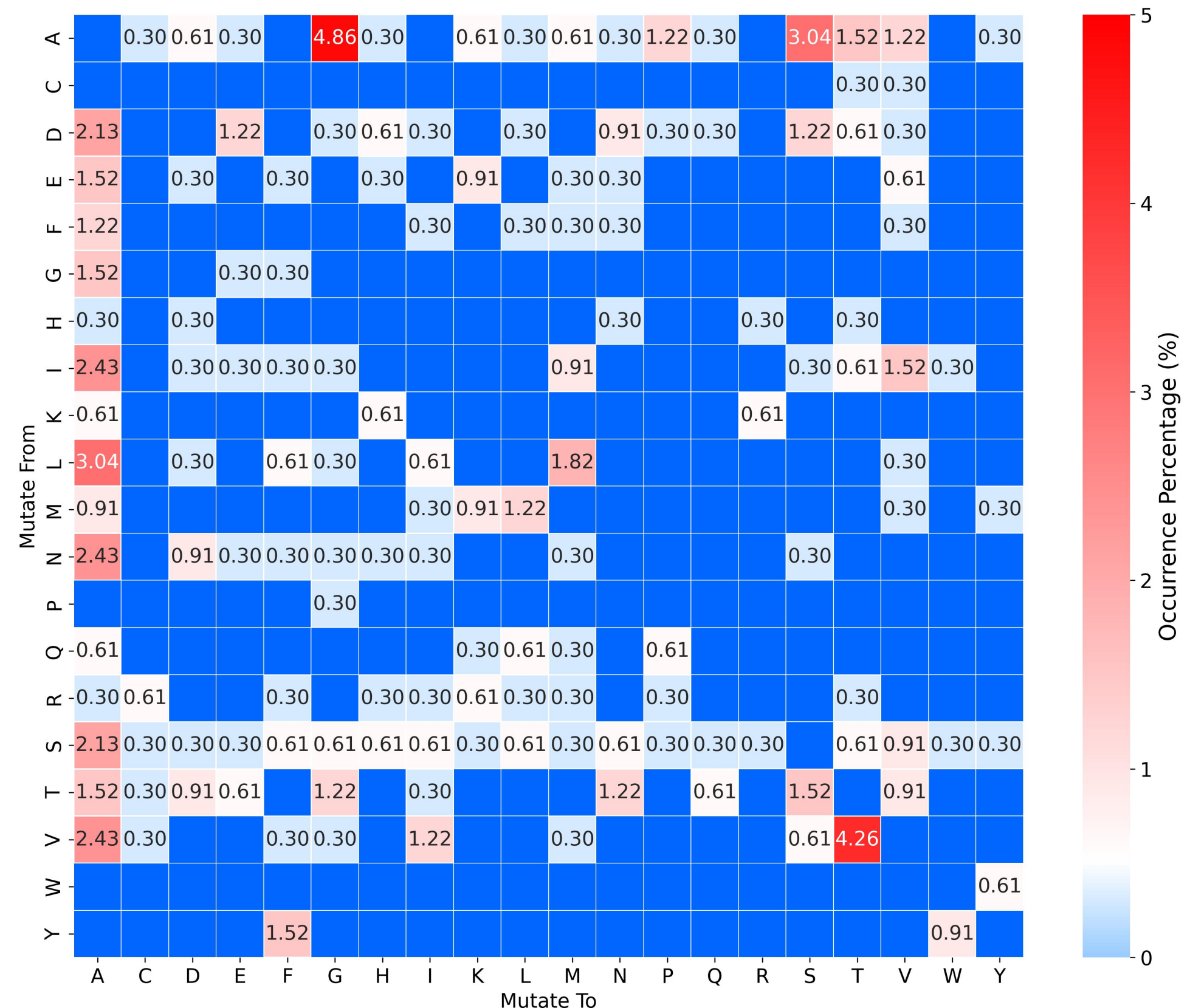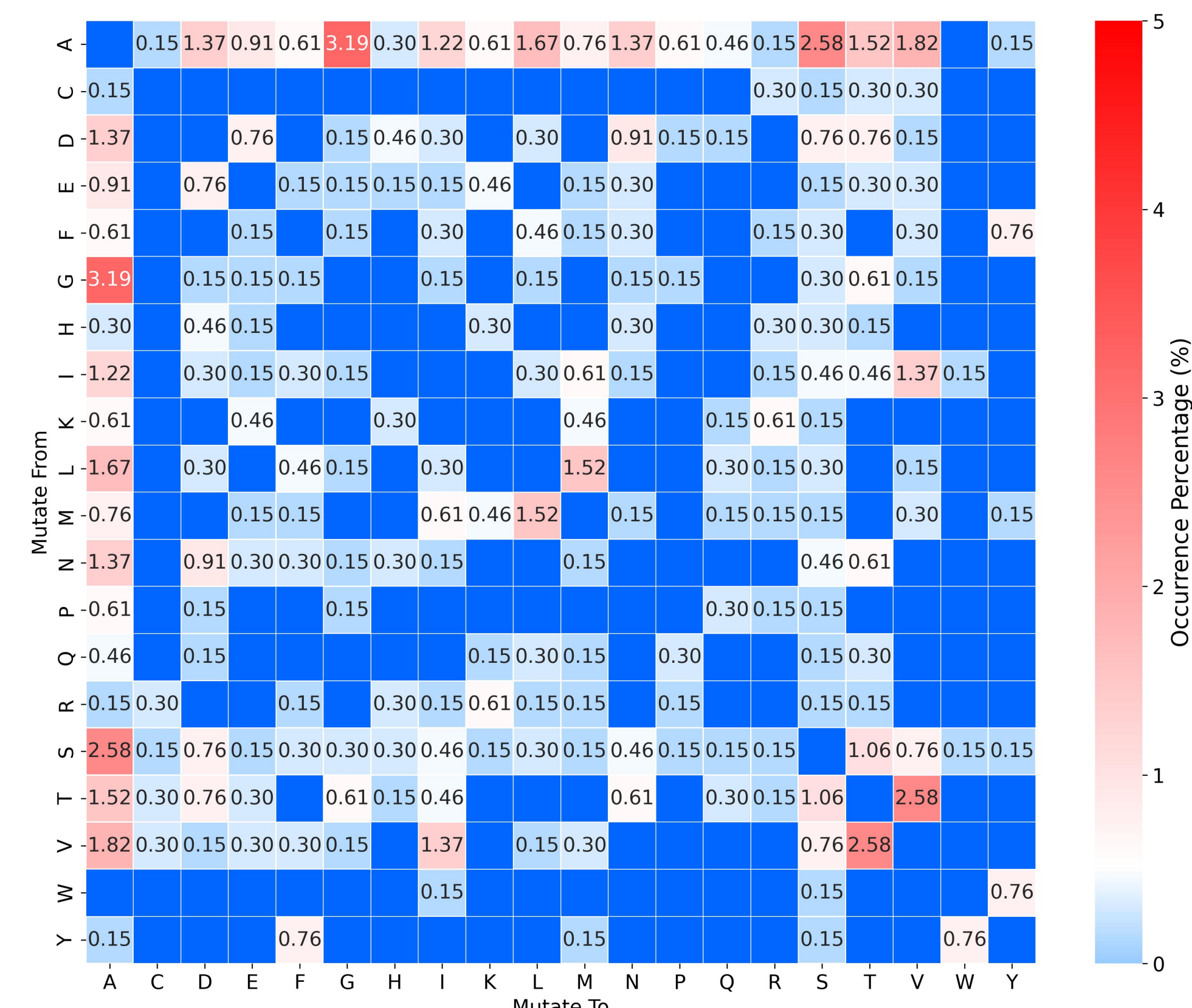

Supplement: Supplementary 1 — Notes S1 to S9 Figs. S1 to S5 Tables S1 to S8 [file research.0674.f1.zip › FigureS1.pdf]

a

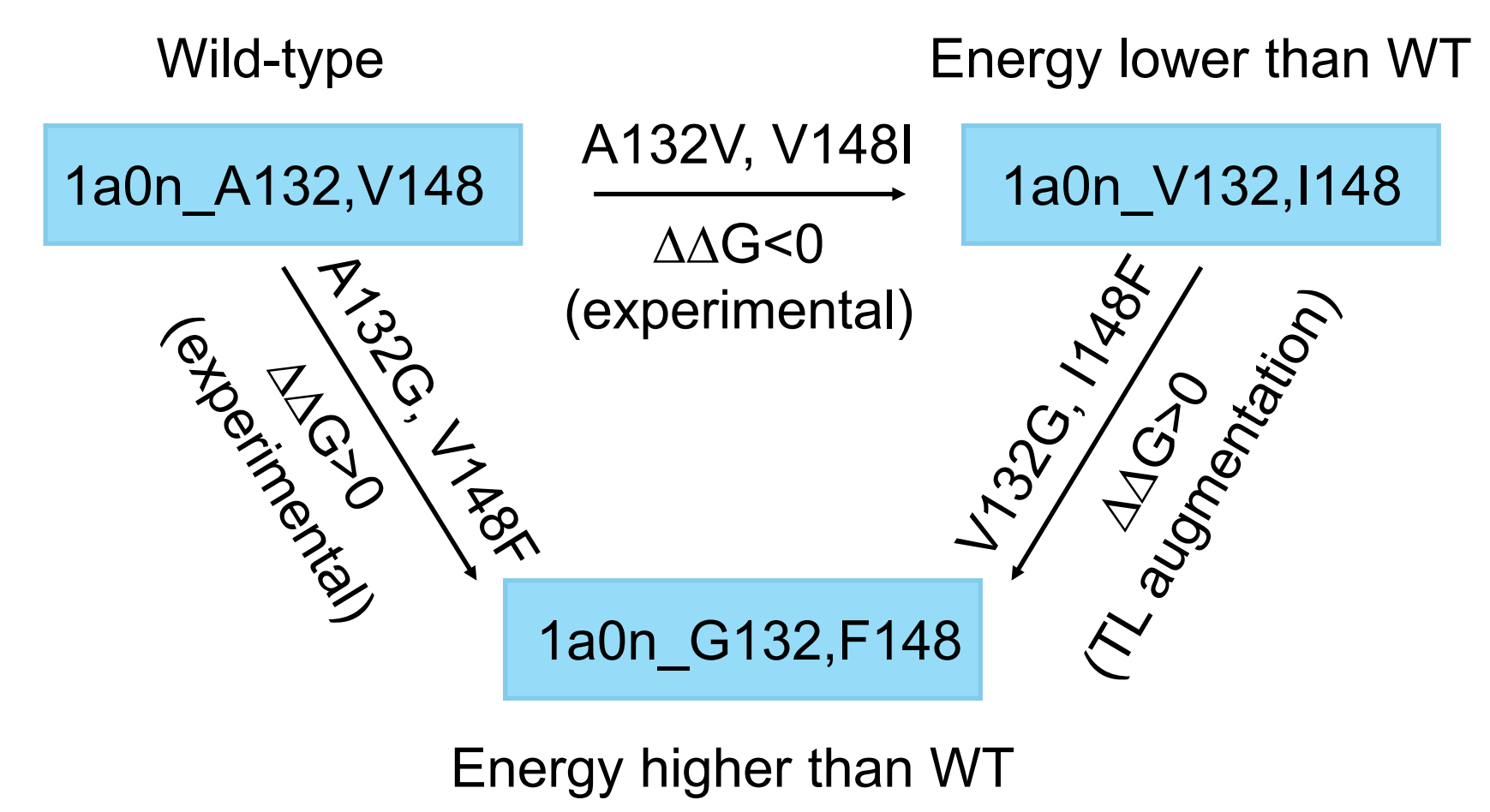

b

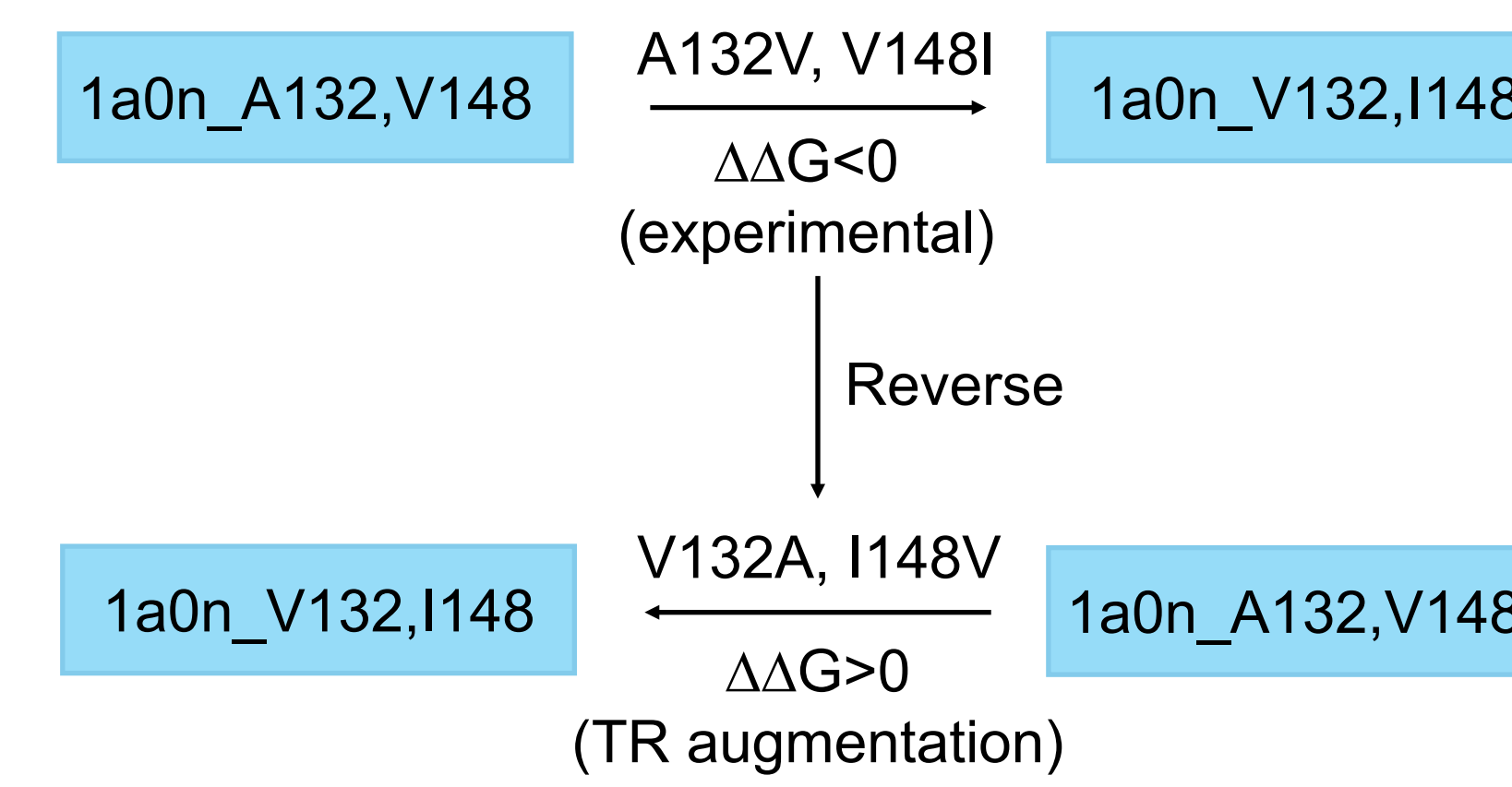

Supplement: Supplementary 1 — Notes S1 to S9 Figs. S1 to S5 Tables S1 to S8 [file research.0674.f1.zip › FigureS2.pdf]

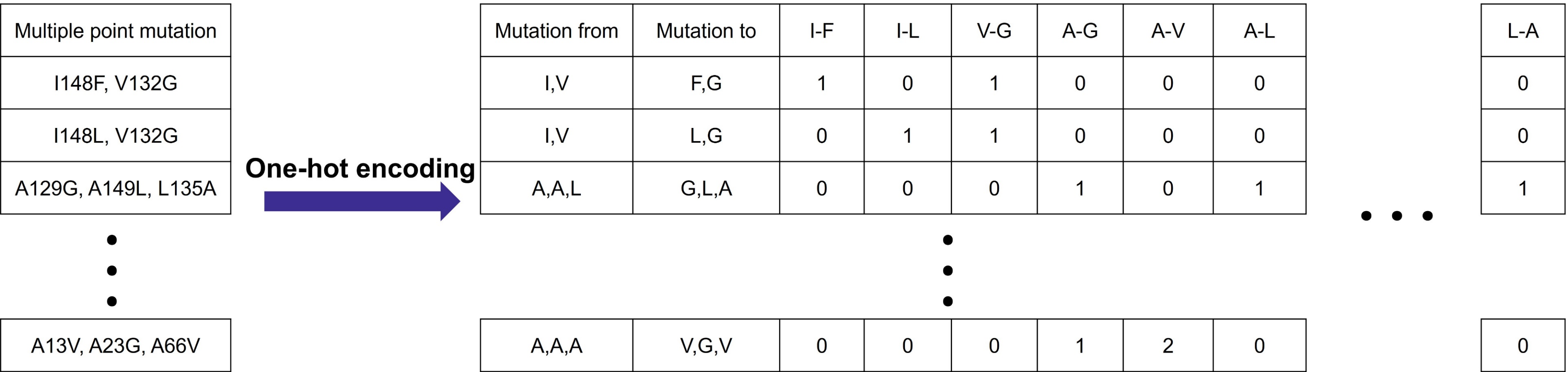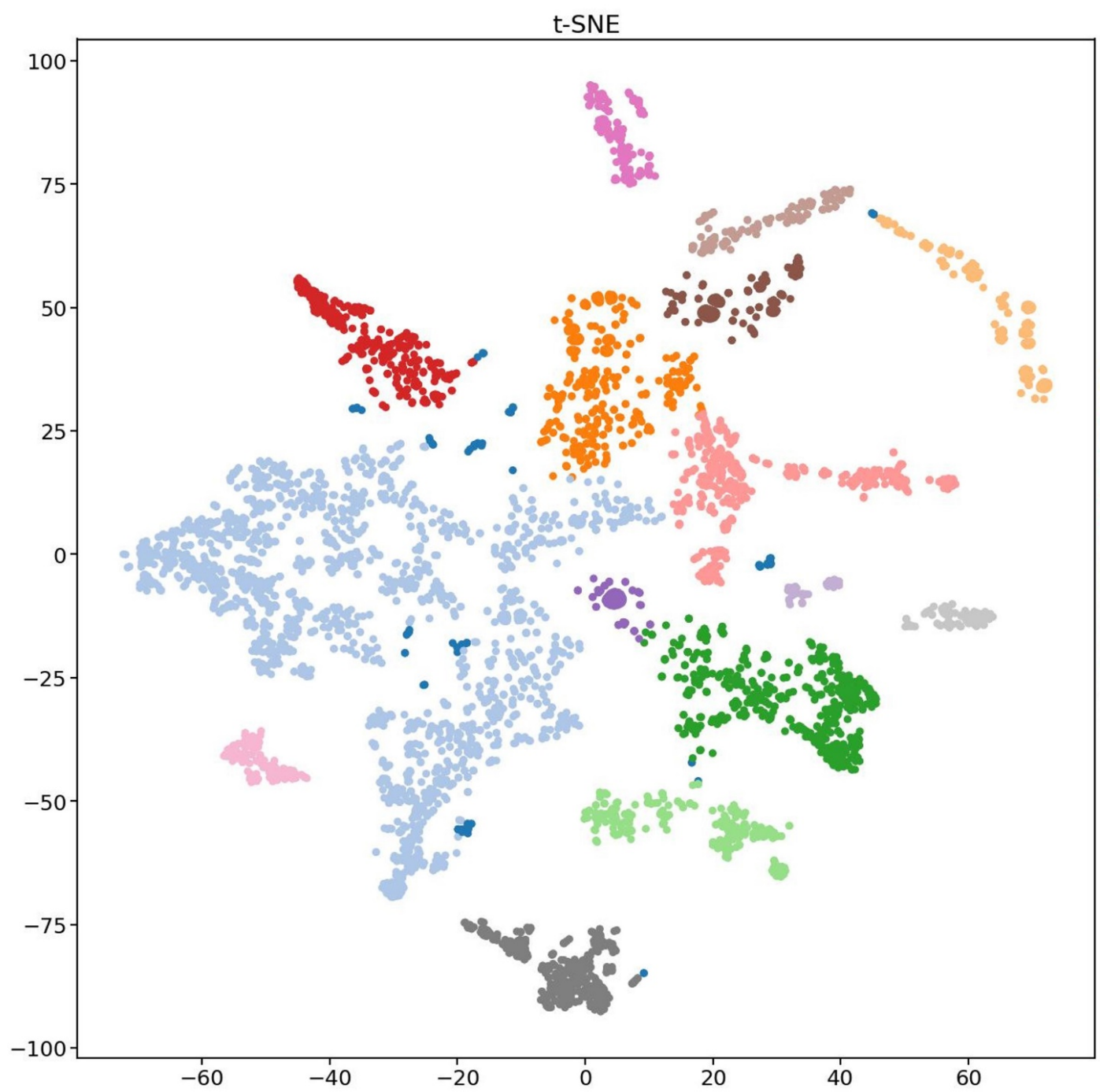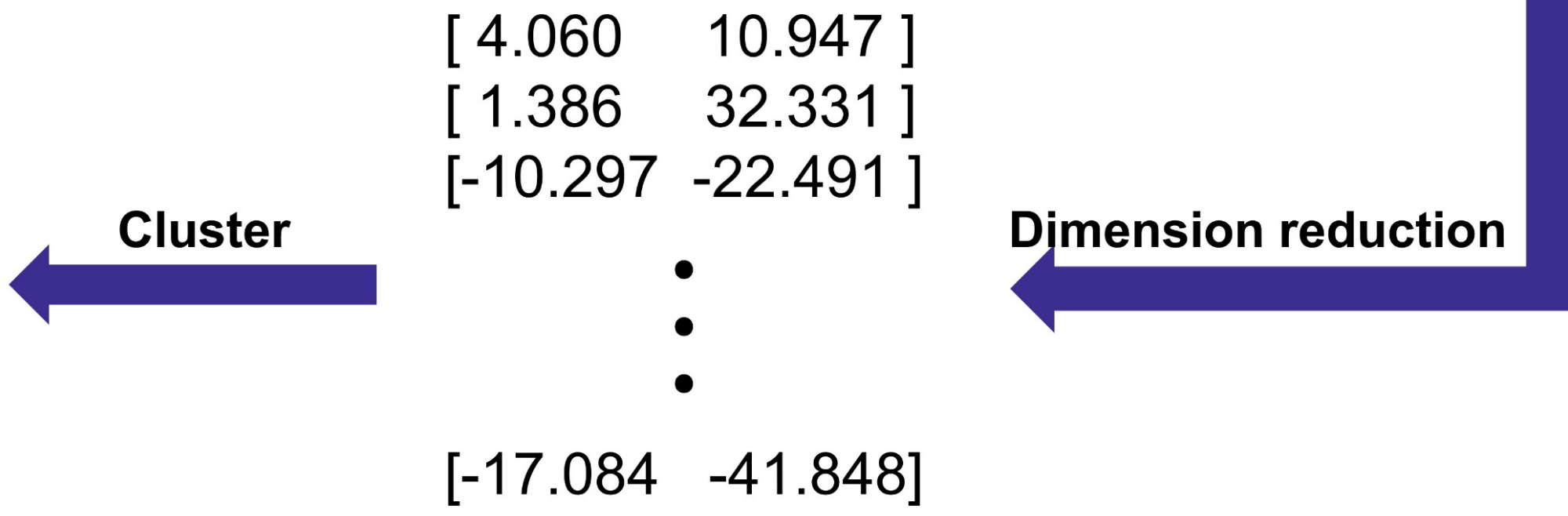

Supplement: Supplementary 1 — Notes S1 to S9 Figs. S1 to S5 Tables S1 to S8 [file research.0674.f1.zip › FigureS3.pdf]

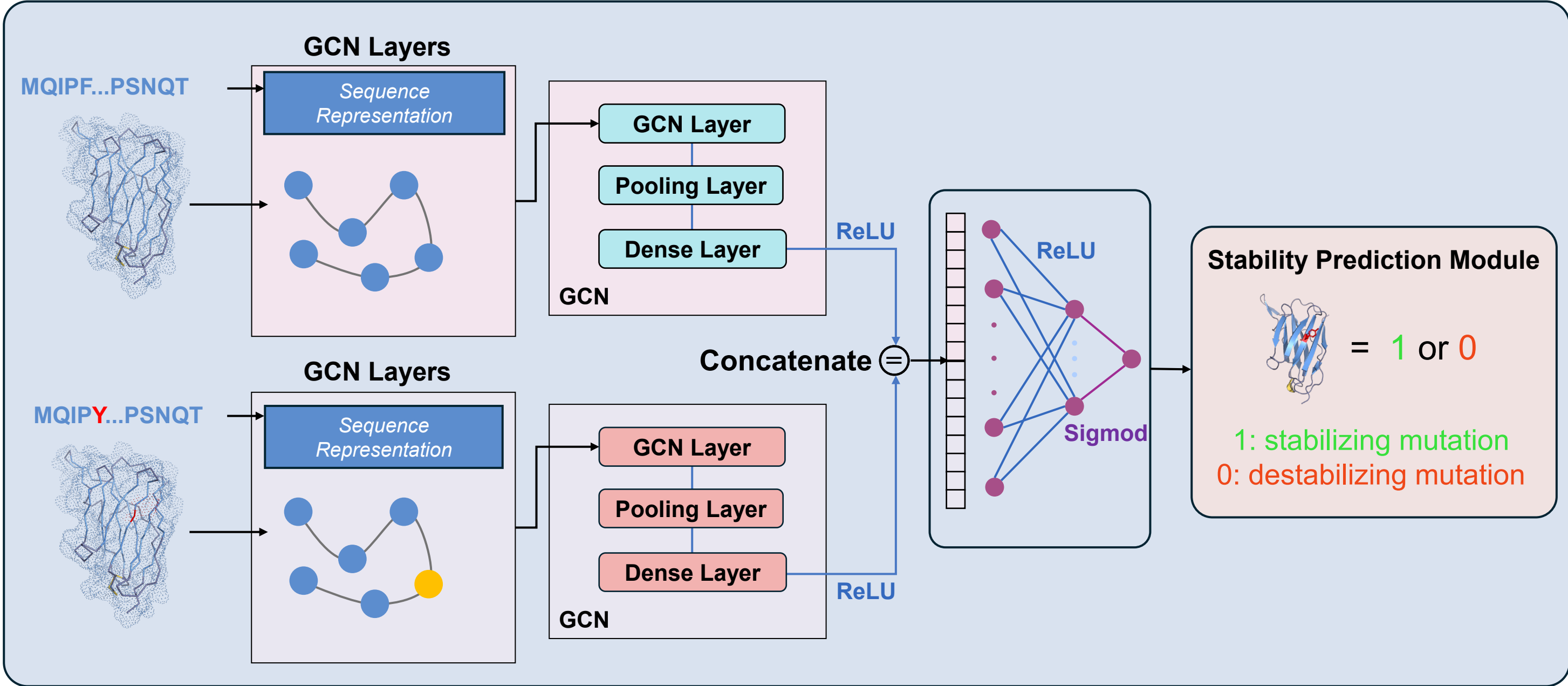

Supplement: Supplementary 1 — Notes S1 to S9 Figs. S1 to S5 Tables S1 to S8 [file research.0674.f1.zip › FigureS4.pdf]
